# Metal‐Supramolecular Drug Delivery System Empowered Meningeal Lymphatic Vessels‐Bridged Intracranial‐Peripheral Dual Immune Modulation for Reversing Glioblastoma Immune Suppression

**DOI:** 10.1002/advs.202522604

**Published:** 2026-02-08

**Authors:** Chenxi Zhang, Zhongsheng Xu, Xiaowen Xu, Zening Zhang, Ranran Luo, Pengchen Ren, Yingying Luo, Qiuchi Wu, Xinyu Liu, Guodong Liu, Xiaojing He, Yun Liu

**Affiliations:** ^1^ Department of Radiology The Second Affiliated Hospital of Chongqing Medical University Chongqing China; ^2^ Department of Neurosurgery The Second Affiliated Hospital of Chongqing Medical University Chongqing China

**Keywords:** glioblastoma, immunotherapy, meningeal lymphatic vessels, tumor‐associated macrophages, vascular endothelial growth factor C

## Abstract

Glioblastoma (GBM) presents significant challenges in treatment due to the presence of the blood‐brain barrier (BBB) and immunosuppressive tumor microenvironment (TME). Here, we developed a novel metal‐supramolecular delivery system (FLM@VC) that empowers meningeal lymphatic vessels (MLVs)‐bridged intracranial‐peripheral dual immune modulation to reverse GBM immune suppression. Using coordination‐driven self‐assembly of lipoic acid (LA), iron ions (Fe^3+^), and bovine albumin (BSA), we engineered nanoassemblies with Verubecestat (MK‐8931) encapsulated and with vascular endothelial growth factor C (VEGF‐C) and c(RGDfK) conjugated. Subcutaneously delivered FLM@VC hijacks the MLVs for brain delivery bypassing the BBB, overcoming the limitations of conventional intravenous administration. Upon tumor accumulation, GSH‐responsive disassembly releases MK‐8931 to reprogram TAMs from the pro‐tumoral M2 to the anti‐tumoral M1 phenotype, thereby eliciting proinflammatory cytokine secretion and enhancing phagocytic clearance of GBM cells. Concurrently, VEGF‐C‐mediated MLV expansion enhances dendritic cell (DC) trafficking to deep cervical lymph nodes (dCLNs), potently priming CD8^+^ T cell responses. This MLVs‐bridged intracranial‐peripheral dual immunomodulation strategy effectively transforms immunologically “cold” GBM into “hot” tumors, resulting in potent tumor eradication and significantly prolonged survival in orthotopic GBM models. It not only presents a novel paradigm for synergistic GBM immunotherapy but also provides an alternative brain drug delivery approach.

## Graphical abstract

A novel metal‐supramolecular nanoplatform (FLM@VC) enables efficient brain delivery via meningeal lymphatic vessels (MLVs), bypassing the blood‐brain barrier (BBB) through subcutaneous administration. It reprograms tumor‐associated macrophages (TAMs) and remodels the MLVs to promote DC trafficking and CD8^+^ T cell activation. This MLVs‐bridged intracranial‐peripheral dual immunomodulation reverses glioblastoma (GBM) immunosuppression, effectively transforming immunologically “cold” GBM into “hot” tumors.

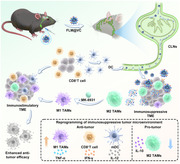

## 1 Introduction

Glioblastoma (GBM) is the most prevalent and aggressive malignant brain tumor in adults, making up approximately 50% of all primary malignant central nervous system (CNS) tumors [1]. Recent developments in GBM treatments, such as surgical treatment, radiation therapy, chemotherapy, and targeted therapy, have not significantly improved the overall prognosis, and long‐term survival is still rare [2]. Therefore, it is crucial to explore better therapeutic options. There have been numerous inspiring clinical advancements as immunotherapy has introduced a different treatment paradigm for malignant tumors. The burgeoning field of nanomedicine has found extensive application in enhancing immunotherapy strategies [3]. Unlike other solid tumors, GBM poses major challenges for immunotherapy due to its unique location in a crucial organ, an immunosuppressive tumor microenvironment (TME), and the blood‐brain barrier (BBB) [1, 4].

**SCHEME 1:**
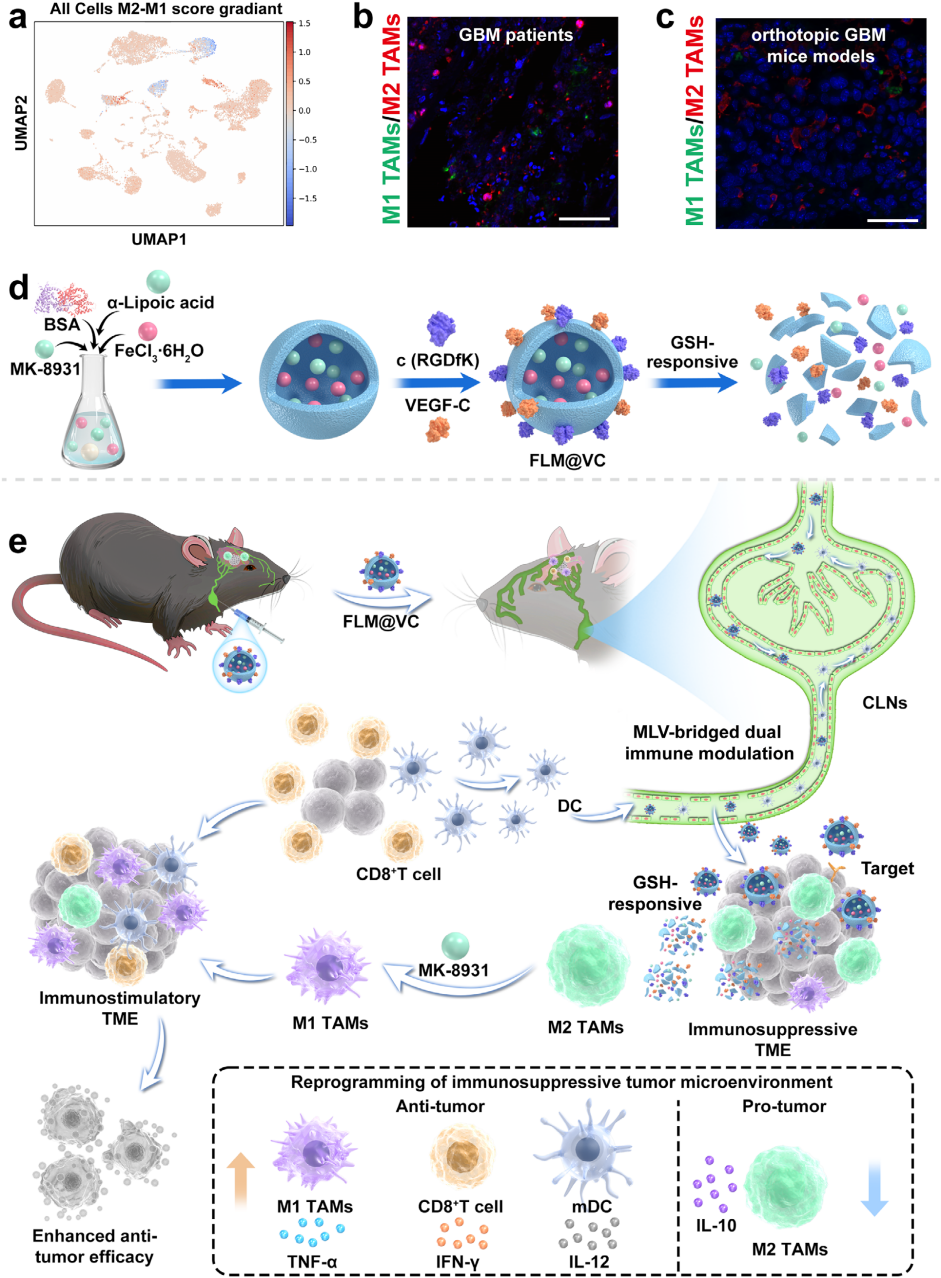
Analysis of TAMs in GBM patients and the schematic illustration of designing FLM@VC to reverse GBM immune suppression. (a) Single‐cell UMAP projection colored by M2‐M1 macrophage polarization score (blue: M2 TAMs; red: M1 TAMs). Representative immunofluorescence images of Arg1 (M2 TAMs) and iNOS (M1 TAMs) expression in tumor tissues resected from (b) GBM patients and (c) orthotopic GBM mice models. Scale bar: 50 µm. Schematic illustration of (d) the fabrication of FLM@VC and (e) how FLM@VC treatment effectively reverses GBM immunosuppression via MLVs‐bridged intracranial‐peripheral dual immune modulation.

GBM is classified as a “cold” tumor due to its infiltration by various immunosuppressive cells, such as tumor‐associated macrophages (TAMs), myeloid‐derived suppressor cells (MDSCs), and regulatory T (Treg) cells [5, 6]. TAMs, the main non‐cancerous immune cells in the GBM microenvironment, comprise 30%–50% of the tumor mass [7]. Bioinformatics analyses of GBM patients revealed a low proportion of M1 TAMs (IL1B, TNF, NOS2, CD86, CXCL9, CXCL10, CCL5, IL12B) and a high proportion of M2 TAMs (MRC1, CD163, ARG1, IL10, CCL18, TGFB1, CD206) in human glioblastoma tissues (Scheme 1a). Meanwhile, our data of GBM patients and orthotopic GBM mice models indicated that the predominance of M2 TAMs was obviously observed in glioblastoma tissues (Scheme 1b,c). Recent studies reveal that M2 TAMs play immunosuppressive roles in the TME and hinder the effectiveness of immunotherapy [8, 9]. Given their role in immune suppression and GBM progression, TAMs are targeted for GBM treatment by preventing their recruitment, depleting them, or reprogramming M2 TAMs to anti‐tumoral M1 TAMs [10, 11]. Notably, β‐site amyloid precursor protein‐cleaving enzyme 1 (BACE1) is preferentially expressed by M2 TAMs, and sustains their protumoral function via activating phosphorylation of signal transducer and activator of transcription 3 (pSTAT3Y^705^) [12]. Pharmacological inhibition of BACE1 disrupts pSTAT3 signaling, inducing the reprogramming of M2 TAMs to M1 TAMs to exert anti‐tumor activity. Mechanistically, BACE1 inhibition disrupted pSTAT3^Y705^ in M2 TAMs, directly triggering their repolarization toward an anti‐tumoral phenotype. This phenotypic conversion potently induced the secretion of proinflammatory cytokines and activated the phagocytic capacity of TAMs, enabling them to engulf tumor cells and trigger anti‐tumor immune responses. Thereby, it transformed the TME from “cold” to “hot”, further enhancing the efficacy of T cell‐based immunotherapy. These findings highlight BACE1 as a promising therapeutic target for macrophage‐based immunotherapy.

However, the effective delivery of nanodrugs to the brain continues to be a major challenge. Previously, different routes have been studied for drug delivery to the brain, such as intravenous, intrathecal, intraparenchymal, and intranasal administration [13]. Intravenous administration is a widely used administration route for nanomedicine administration due to its noninvasive nature, procedural simplicity, and suitability for repeated dosing. Nevertheless, the limited permeability of the BBB, metabolism by the liver and kidneys, and interception by the reticuloendothelial system (RES) may limit its efficiency [14, 15]. Intrathecal administration bypasses the BBB by directly delivering nanodrugs into the cerebrospinal fluid (CSF) and then to the CNS, though its invasiveness limits use to specific types of medical conditions within the central nervous system [16, 17, 18]. Intraparenchymal administration, which delivers nanodrugs directly to diseased sites, offers superior targeting precision but comes with higher invasiveness and surgical risks [19]. Intranasal administration provides a noninvasive alternative to bypass the BBB. However, limitations such as nasal passage conditions and potential drug entrapment within the olfactory mucosa may reduce particle retention time and hinder migration into the CNS [20, 21]. Therefore, there is a clear need for better drug delivery routes to improve the therapeutic efficacy of GBM.

Emerging evidence has highlighted meningeal lymphatic vessels (MLVs) as a promising therapeutic conduit for drug delivery to the brain [22]. The CNS is traditionally viewed as immune‐privileged because of limited immune surveillance, a consequence of the BBB and the lack of a specific lymphatic system [23, 24]. However, this perspective has been transformed by the discovery of MLVs. MLVs, situated in the dura mater adjacent to the dural sinuses, perform traditional lymphatic functions such as drainage and immunoregulation. These vessels bridge the CNS and peripheral lymphatic networks, enabling immune cell trafficking [25, 26]. Thus, MLVs are crucial for linking the intracranial and peripheral immune systems. Previous studies have confirmed the feasibility of this delivery route [22, 27, 28]. Vascular endothelial growth factor C (VEGF‐C), identified as a lymphangiogenesis factor, drives the remodeling of MLVs, enhancing their drainage function to facilitate brain drug delivery [29, 30]. This VEGF‐C‐induced MLV remodeling promotes C‐C motif chemokine ligand 21 (CCL21)‐dependent migration of DCs to deep cervical lymph nodes (dCLNs). Within these dCLNs, DCs stimulate the proliferation of tumor‐infiltrating CD8^+^ T cells, thereby amplifying anti‐tumor immune responses [31]. This VEGF‐C‐mediated enhancement of MLV function and subsequent priming of anti‐tumor T cell responses support a promising therapeutic role for VEGF‐C in augmenting immunotherapy for GBM.

Herein, we proposed an intelligent metal‐supramolecular delivery system (termed FLM@VC) to empower intracranial‐peripheral dual immune modulation against GBM. As depicted in Scheme 1d,e, the nanosystem was fabricated via a stepwise coordination‐driven self‐assembly process involving lipoic acid (LA), ferric ions (Fe^3+^), and bovine serum albumin (BSA). Initially, Fe^3+^ chelated with BSA, subsequently triggering the disulfide ring‐opening polymerization of LA and guiding its self‐assembly into a well‐defined nanostructure, which was stabilized by the strong coordination between LA and Fe^3+^. In FLM@VC construction, BACE1 inhibitor Verubecestat (MK‐8931) was encapsulated, while the c(RGDfK) peptides and VEGF‐C were conjugated. Following subcutaneous injection in the neck site, nanoparticles accumulated in dCLNs and continuously diffused into the brain via the MLVs, bypassing the BBB. The c(RGDfK) peptides then bound to integrin αvβ3, which is overexpressed on tumor cells, thereby enhancing tumor‐specific delivery. At the tumor site, the disulfide bonds in the nanostructure were cleaved by GSH, leading to framework disassembly and the release of MK‐8931. MK‐8931 repolarized TAMs toward an anti‐tumoral phenotype, eliciting proinflammatory cytokine secretion and enhancing phagocytic clearance of GBM cells, thereby activating the intracranial immune response. Meanwhile, VEGF‐C promoted MLV remodeling, thereby potentiating DC trafficking and subsequent CD8^+^ T cell activation to prime peripheral immune response. By synergistically integrating intracranial TME reshaping with peripheral VEGF‐C‐driven MLV remodeling and T cell recruitment, this dual‐immunotherapy empowered FLM@VC to effectively convert the immunosuppressive TME into an immunostimulatory niche, leading to robust anti‐tumor activity. This work offers novel insights for GBM immunotherapy and paves the way for exploring alternative approaches for the delivery to the brain.

## 2 Results and Discussion

### 2.1 Preparation and Characterization of FLM@VC

**FIGURE 1:**
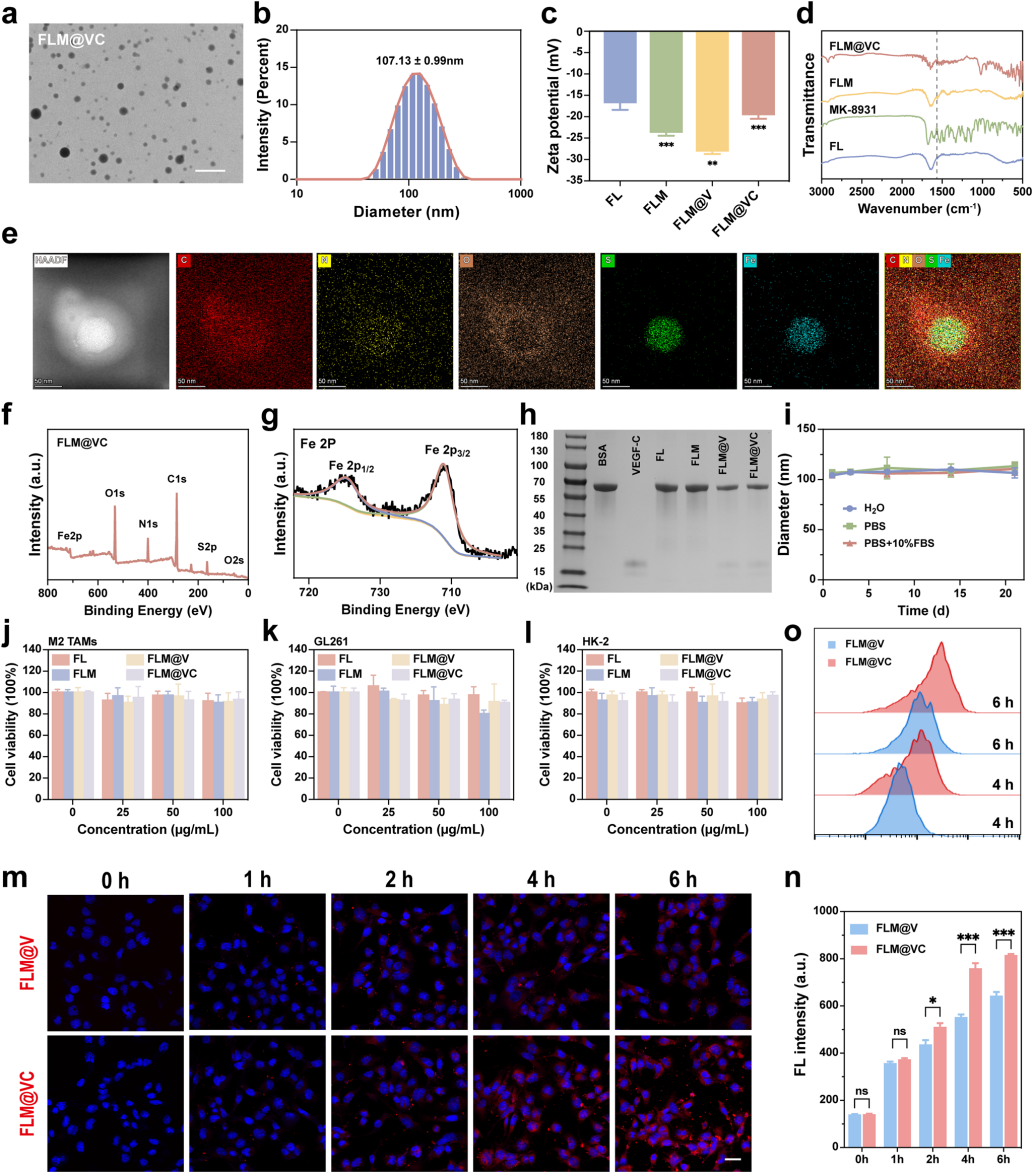
Characterization, cytotoxicity, and intracellular uptake of FLM@VC. (a) TEM of FLM@VC, Scale bar: 250 nm. (b) DLS of FLM@VC. (c) Zeta potential of FL, FLM, FLM@V, and FLM@VC. (d) FTIR spectra of FL, MK‐8931, FLM, and FLM@VC. (e) Elemental mapping image of FLM@VC. Scale bar: 50 nm. (f) XPS survey spectra and (g) XPS spectra of Fe2p. (h) SDS‐PAGE assay of FL, FLM, FLM@V, and FLM@VC. (i) DLS of FLM@VC in different solutions. (j–l) Cell viability of M2 TAMs, GL261 cells, and HK‐2 cells after incubation with FL, FLM, FLM@V, and FLM@VC. Data are shown as the mean values ± SD (n = 3). (m, n) Intracellular uptake of Cy5‐labeled FLM@V and FLM@VC under CLSM and the corresponding statistical analysis. Scale bar: 25 µm. Data are shown as the mean values ± SD (n = 3). (o) FCM analysis of GL261 cells after different incubation times with Cy5‐labeled FLM@V and Cy5‐labeled FLM@VC. All the statistical significance was analyzed by two‐tailed Student's t‐test, *p < 0.05, **p < 0.01, ***p < 0.001, and ****p < 0.0001, ns, not significant.

The fabrication procedure was shown in Scheme 1d. FLM@VC NPs were constructed using a self‐assembly method. To confirm the coordination‐driven assembly process among LA, Fe^3+^, and BSA, we first characterized the intermediate product BSA‐Fe^3+^ and the final assembled nanoparticle FL. As shown in Figure S1a, BSA‐Fe^3+^ complex appeared as dispersed, irregular aggregates, while FL exhibited well‐defined spherical nanostructures. Elemental mapping of FL demonstrated uniform distribution of C, N, O, S, and Fe (Figure S1b), confirming the successful integration of LA, Fe^3+^, and BSA into a metal‐supramolecular framework via coordination interactions. Moreover, transmission electron microscopy (TEM) and high‐resolution transmission electron microscopy (HRTEM) demonstrated that the FLM@VC NPs were spherical with an average size of ∼50 nm (Figure 1a; Figure S2). Then, the mean hydrodynamic diameter of FLM@VC NPs was 107 nm using dynamic light scattering (DLS) (Figure 1b). The zeta potentials of FL, FLM, FLM@V, and FLM@VC NPs were all negative, among which FLM@V and FLM@VC NPs were −28 and −18 mV, indicating the successful conjugation of c(RGDfK) peptides (Figure 1c). The Fourier‐transform infrared spectroscopy (FTIR) results (Figure 1d) revealed the stretching vibrations of ‐CONH‐ at 1568 cm^−1^ in the absorption bands of FLM@VC NPs, confirming the presence of MK‐8931. Elemental mapping revealed the presence of C, N, O, S, and Fe (Figure 1e). Moreover, the elementary composition and metallic oxidation state of the Fe species in FLM@VC NPs were examined by X‐ray photoelectron spectroscopy (XPS). As shown in Figure 1f, the FLM@VC NPs were mainly composed of C, N, O, S, and Fe. In addition, the as‐prepared FLM@VC showed clear Fe 2p_1/2_ and 2p_3/2_ photoelectron peaks at binding energies of 724.63 and 710.99 eV (Figure 1g), which confirmed that the FLM@VC NPs were composed mainly of trivalent iron ions. Furthermore, surface protein on FLM@VC was analyzed using sodium dodecyl sulfate‐polyacrylamide gel electrophoresis (SDS‐PAGE). The similar protein profiles of VEGF‐C, FLM@V, and FLM@VC NPs demonstrated that the VEGF‐C was preserved after incorporation into the FLM NPs (Figure 1h). In addition, the DLS results showed that the diameter of FLM@VC in H_2_O, PBS, and PBS with a 10% FBS solution remained stable for 21 d (Figure 1i), suggesting the long‐term stability of FLM@VC NPs in a biological environment. To assess the stability and morphological changes of nanoparticles after response, we dissolved FLM@VC NPs in H_2_O with and without 5 mm GSH, respectively. After 6 h, their morphological changes were recorded via TEM images (Figure S3). FLM@VC displayed spherical morphology in H_2_O, but in 5 mm GSH, the NPs were larger and formed amorphous aggregates with a more complex background. As demonstrated in Figures S4–S6, less than 20% of VEGF‐C, c(RGDfK) or MK‐8931 was released in H_2_O over 24 h, whereas over 80% was released in the presence of 5 mm GSH, indicating that FLM@VC NPs have excellent GSH‐responsive abilities and the potential for GSH‐triggered drug release.

Following the characterization of their physicochemical properties and stability, the biosafety and targeting capability of FLM@VC NPs were then assessed at the cellular level. We first investigated the cytotoxicity of FL, MK‐8931, FLM, FLM@V, and FLM@VC NPs on IL‐4‐activated RAW264.7 cells (M2 TAMs), GL261 cells, and HK‐2 cells. The CCK‐8 assay results showed that M2 TAMs and GL261 cells remained viabilities above 80% after 24 h of incubation with all NPs (Figure 1j–l), indicating no significant cytotoxicity at concentrations ranging from 25 to 100 µg mL^−1^. As shown in Figure S7, at MK‐8931 concentrations ranging between 1.25 and 5 µg mL^−1^, the viabilities of M2 TAMs, GL261 cells, and HK‐2 cells remained above 80%. Therefore, in subsequent experiments, 100 µg mL^−1^ of the FL, FLM, FLM@V, and FLM@VC NPs and 5 µg mL^−1^ of MK‐8931 were used to evaluate the targeting of FLM@VC, the mechanism of TAMs polarization, and the antitumor effects in vitro. In addition, the tumor‐targeting capability of FLM@VC NPs was evaluated based on GL261 cells uptake. We subsequently labeled FLM@V and FLM@VC NPs with Cy5 and incubated them with GL261 cells. Confocal laser scanning microscopy (CLSM) images showed a time‐dependent increase in fluorescence intensity for both Cy5‐labeled FLM@V and FLM@VC over 6 h (Figure 1m,n, Figure S8). Notably, Cy5‐labeled FLM@VC exhibited significantly higher tumor cell‐targeting efficiency than Cy5‐labeled FLM@V, particularly after incubation for 4 and 6 h. Flow cytometry (FCM) analysis further supported this result (Figure 1o), indicating that the c(RGDfK) peptide modification enhanced tumor‐specific delivery by targeting the highly expressed integrin ανβ3 in GL261 cells.

Collectively, the FLM@VC was fabricated and characterized successfully. With spherical structures, favorable nanoscale size, GSH responsiveness, and biocompatibility, FLM@VC also exhibited efficient targeting capability, making FLM@VC a promising candidate for targeted GBM treatment.

### 2.2 FLM@VC Promotes TAM Repolarization via Inhibiting the BACE1‐STAT3 Signaling Pathway in Vitro

**FIGURE 2:**
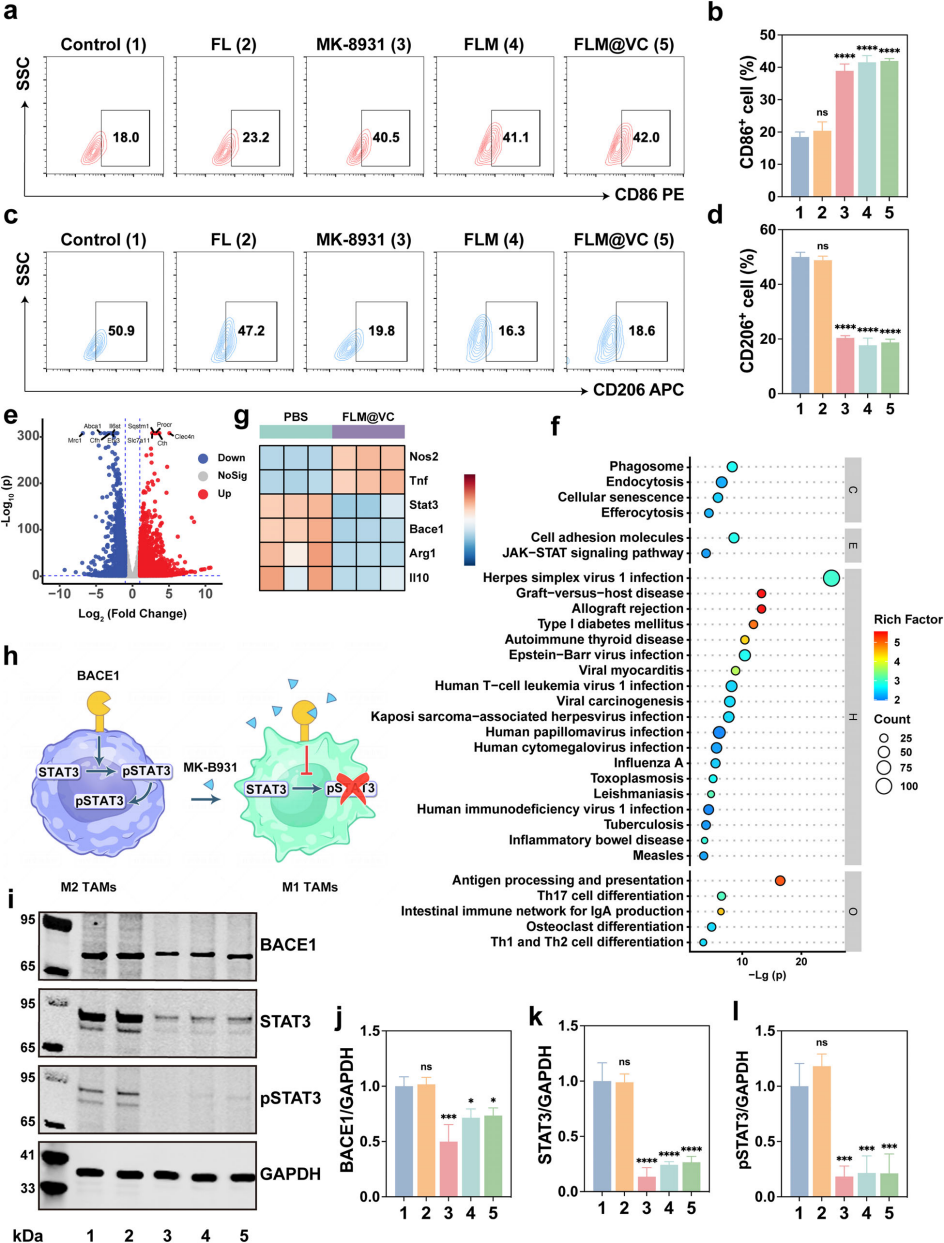
Investigation of macrophage polarization in vitro. FCM analysis of (a, b) CD86^+^ and (c, d) CD206^+^ expression in RAW264.7 cells following different treatments. Data are represented as mean ± SD (n = 3). (e) Volcano plot of genes upregulated or downregulated among the differentially expressed genes in FLM@VC and the Control group. (f) The enriched pathways analysis. (g) heat map. (h) A schematic diagram illustrates how MK‐8931 inhibits BACE1, reprogramming M2 TAMs to M1 TAMs. (i–l) WB analysis of key protein expression and the corresponding quantitative analysis. Data are shown as the mean values ± SD (n = 3). All the statistical significance was analyzed by ANOVA with a Tukey post‐hoc test. *p < 0.05, **p < 0.01, ***p < 0.001, and ****p < 0.0001, ns, not significant.

To evaluate the potential of FLM@VC to exert macrophage‐mediated immunotherapy, we next investigated the ability of MK‐8931, FLM, and FLM@VC to repolarize pro‐tumoral M2 TAMs into the anti‐tumoral M1 TAMs that can participate in antitumorigenic activities. M2 TAMs were treated with PBS, FL, MK‐8931, FLM, or FLM@VC for 24 h. FCM analysis was performed to evaluate the expression of CD86 (M1 marker) and CD206 (M2 marker). Compared to the Control group (CD206^+^: 50.9%, CD86^+^: 18.0%), the FL group (CD206^+^: 47.2%, CD86^+^: 23.2%) showed no significant difference (Figure 2a–d), indicating that the FL NPs alone merely serve as a delivery vehicle without therapeutic effect. In contrast, the MK‐8931, FLM, and FLM@VC groups all exhibited a substantial shift toward the M1 phenotype, with the percentage of CD206^+^ cells decreasing to 19.8%, 16.3%, and 18.6% and the percentage of CD86^+^ cells increasing to 40.5%, 41.1%, and 42.0%, respectively (Figure 2a–d). These results demonstrated that MK‐8931 could repolarize M2 TAMs to M1 TAMs, consistent with previous reports [12]. Meanwhile, nanoparticle formulation modifications (FLM NPs and FLM@VC NPs) maintained drug efficacy comparable to free MK‐8931. The above results highlighted the potent effect of FLM@VC NPs in repolarizing M2 TAMs for immunotherapy.

To further explore the underlying mechanism of the repolarization of M2‐to‐M1 TAMs, RNA transcription analysis was performed on M2 TAMs subjected to FLM@VC treatment and Control conditions. We found that expression of 3309 genes (1709 genes upregulated and 1600 genes downregulated) was altered by FLM@VC treatment (Figure 2e; Figure S9). By comparing the FLM@VC group and the untreated group, Gene Ontology (GO) annotation analysis showed that most of the differential genes were concentrated in “cellular processes,” “biological regulation,” “binding”, “transcription regulation,” and other processes (Figure S10). Moreover, Kyoto Encyclopedia of Genes and Genomes (KEGG) analysis revealed enrichment of pathways associated with immunoregulatory functions, including “JAK‐STAT3 signaling pathway”, and “antigen processing and presentation” (Figure 2f). Importantly, genes related to M1 TAMs were upregulated, while most M2 TAMs‐related genes were downregulated in the FLM@VC group (Figure 2g), indicating its ability to shift M2 TAMs toward an M1 phenotype. Critically, this included substantial suppression of the key regulators BACE1 and STAT3. Mechanistically, BACE1 sustains M2 polarization through proteolytic generation of soluble mediators that trigger STAT3 phosphorylation. Activated STAT3 then translocates to the nucleus, driving transcription of pro‐tumorigenic factors that reinforce immunosuppressive functions. FLM@VC‐mediated disruption of this BACE1‐STAT3 axis functionally reprogrammed TAMs toward anti‐tumoral M1 phenotypes. The schematic diagram of BACE1‐STAT3 signaling inhibition was displayed in Figure 2h. Finally, to further investigate the potential mechanism underlying the anti‐GBM effects, the changes in the expression of key signaling pathway proteins were examined using western blot (WB). BACE1 is preferentially expressed by M2 TAMs and maintains their pro‐tumorigenic state through STAT3‐dependent signaling. Figure 2i,j revealed a reduction in BACE1 protein expression in the MK‐8931, FLM, and FLM@VC groups compared to the Control and FL groups, indicating that MK‐8931 facilitated M2‐to‐M1 repolarization via BACE1 inhibition. To elucidate how BACE1 maintains M2 TAMs, we focused on STAT3, a key transcriptional regulator of macrophage polarization, and investigated its role in this process. Statistical analysis revealed a significant reduction in the expression ratio of STAT3 and pSTAT3 proteins in the MK‐8931, FLM, and FLM@VC groups compared to the Control and FL groups (Figure 2i,k,l), indicating that the FLM and FLM@VC NPs successfully inhibited the BACE‐STAT3 signaling pathway.

These findings demonstrated that FLM@VC NPs effectively repolarized M2 TAMs toward anti‐tumoral M1 TAMs, as evidenced by increased CD86 and decreased CD206 expression. This functional shift resulted from FLM@VC potently inhibiting the BACE1‐STAT3 pathway. By downregulating BACE1, FLM@VC suppressed STAT3 expression and its phosphorylation, disrupting its BACE1‐dependent activation and the pro‐tumoral M2 phenotype it sustains.

### 2.3 TAM Repolarization Triggers Anti‐tumor Effects in Vitro

**FIGURE 3:**
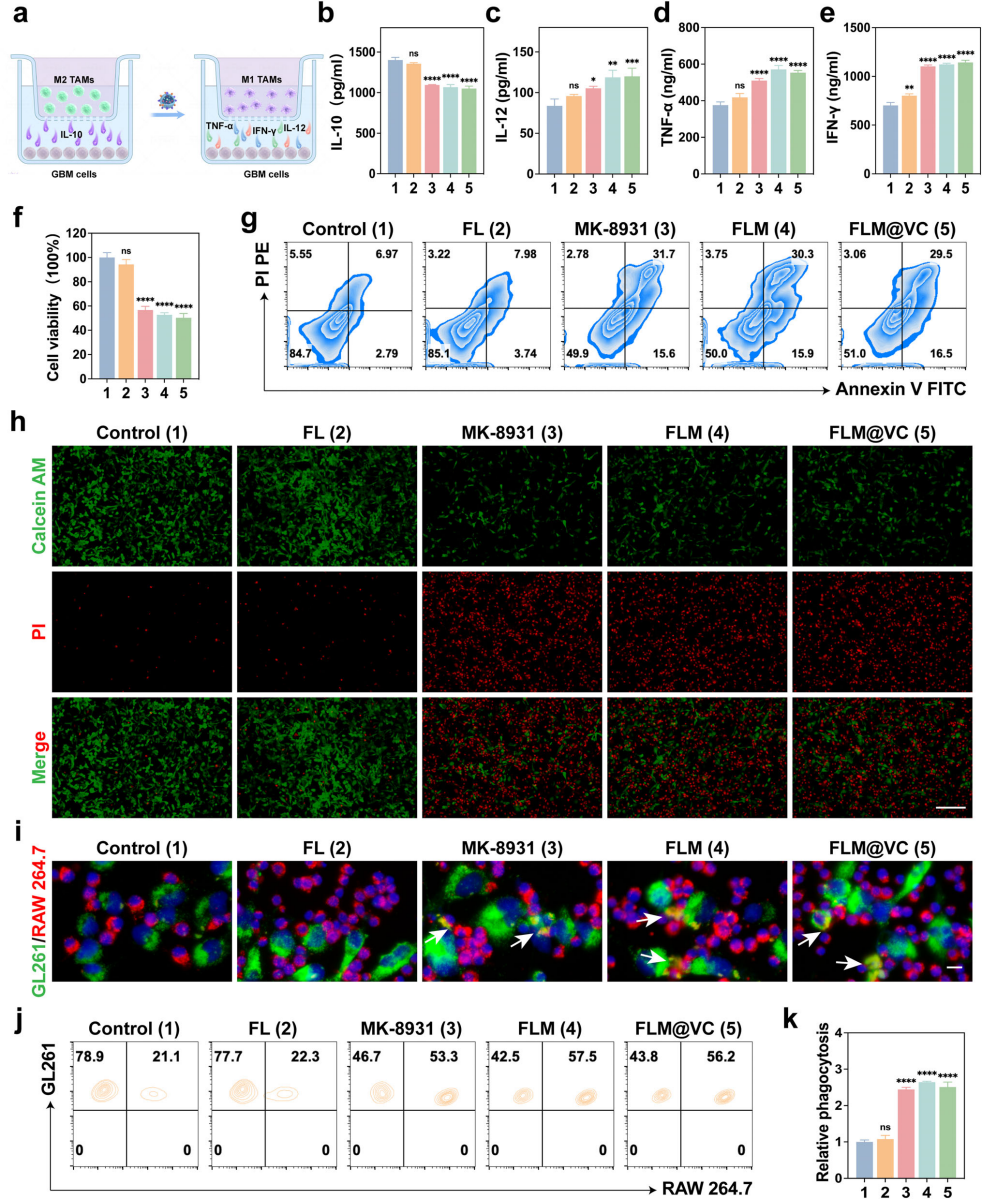
In vitro antitumor effect of FLM@VC. (a) Illustration of the co‐culture system involving GL261 cells and M2 TAMs. (b–e) ELISA test of the secretion level of and IL‐10, IL‐12, TNF‐α, and IFN‐γ in the coculture system following different treatments. Data are shown as the mean values ± SD (n = 3). (f) Cell viability of GL261 cells after various treatments. Data are shown as the mean values ± SD (n = 3). (g) FCM analysis of GL261 cells apoptosis after various treatments. (h) Live/dead cells stained with Calcein‐AM (green)/PI (red) in a fluorescence microscope. Scale bar: 100 µm. (i) Representative images for analyzing the killing of GL261 tumor cells by the repolarized M1 macrophages, with arrows indicating phagocytosis of tumor cells by the macrophages (white). Scale bar: 25 µm. (j–k) FCM analysis of macrophage phagocytosis of GL261 cells in vitro. Data are shown as the mean values ± SD (n = 3). All the statistical significance was analyzed by ANOVA with a Tukey post‐hoc test. *p < 0.05, **p < 0.01, ***p < 0.001, and ****p < 0.0001, ns, not significant.

Emerging evidence has underscored the therapeutic potential of M1 TAMs in orchestrating anti‐tumor immunity through a multifaceted immunostimulatory program. Central to this process is their IFN‐γ‐driven pro‐inflammatory phenotype, characterized by the secretion of key cytokines such as IL‐12, TNF‐α. These cytokines not only perpetuate local inflammatory cascades but also directly enhance the activation and effector functions of cytotoxic CD8^+^ T lymphocytes. This cytokine‐mediated crosstalk effectively bridges innate and adaptive immunity, fostering the establishment of an immunostimulatory TME. Beyond their secretory profile, M1 TAMs exhibit dual functional attributes that include heightened phagocytic capacity enabling efficient clearance of malignant cells and apoptotic debris, alongside potent antigen‐presenting capabilities. Furthermore, they provide co‐stimulatory signals and cytokines that critically support CD8^+^ T cell cytotoxicity. The integration of phagocytic clearance, T cell activation, and immune cell potentiation creates a synergistic anti‐tumor response in which tumor cell elimination is coupled with the amplification of anti‐tumor immunity. Collectively, these mechanisms rationalize the development of therapeutic strategies leveraging M1 TAMs polarization to enhance immunotherapy outcomes. Based on the effective polarization effect of the FLM@VC NPs on TAMs, a transwell co‐culture system was employed to explore the effects of their mediated macrophage repolarization on the efficacy against GBM (Figure 3a). Enzyme‐linked immunosorbent assay (ELISA) analysis demonstrated that FLM@VC NPs (Figure 3b) decreased anti‐inflammatory IL‐10 secretion and (Figure 3c–e) increased pro‐inflammatory IL‐12, TNF‐α, and IFN‐γ secretion compared to the Control group, suggesting the enhanced reprogramming capability of FLM@VC. CCK‐8 assays showed that MK‐8931, FLM, and FLM@VC treatment of M2 TAMs significantly reduced the viability of co‐cultured GL261 cells to 57%, 53%, and 50%, respectively, compared to the Control group after 24 h (Figure 3f). While FL treatment (94%) showed no significant difference, the potent tumor cell killing effects observed with MK‐8931, FLM, and FLM@VC were comparable among these groups. This enhanced cytotoxicity was attributed to MK‐8931‐mediated polarization of TAMs toward an anti‐tumoral M1‐like phenotype. Critically, encapsulation within the FLM and FLM@VC nanoplatforms effectively retained the polarization‐modulating effects and anti‐tumor efficacy of MK‐8931, suggesting these nanocarriers do not compromise its pharmacological activity. Furthermore, such nanoformulations hold significant promise for enhanced therapeutic potential in vivo, potentially offering improved safety profiles compared to free drug administration. According to the results of FCM, the FLM@VC NPs induced significantly higher apoptosis compared to the Control and FL groups (Figure 3g). The results of Calcein AM/PI staining were in line with the results of FCM (Figure 3h), further proving that the FLM@VC NPs were able to significantly induce apoptosis of tumor cells.

We next examined the phagocytosis of TAMs repolarized from M2 to M1 by exposing M2 TAMs to different treatments and co‐culturing them with GL261 cells. Fluorescence microscope imaging showed FLM@VC NPs‐treated M2 TAMs exhibited stronger phagocytosis of GL261 cells (Figure 3i; Figure S11). Moreover, FCM analysis further confirmed these observations (Figure 3j,k). Compared to the Control group, M2 TAMs treated with FL showed minimal enhancement (∼1.1‐fold). Significant increases in phagocytosis were observed in the MK‐8931 (∼2.4‐fold), FLM (∼2.6‐fold), and FLM@VC (∼2.5‐fold) groups. The FCM data demonstrated that both MK‐8931 alone and the nanoparticle formulations were highly effective in reprogramming M2 TAMs toward M1 TAMs, resulting in a substantial (∼2.4–2.6‐fold) increase in GL261 cell engulfment compared to the Control and FL groups.

These results indicated that FLM@VC NPs enhanced antitumor efficacy by reprogramming M2 TAMs to M1 TAMs, stimulating proinflammatory cytokine secretion and promoting phagocytosis of GL261 cells by TAMs, which collectively inhibited tumor growth.

To further evaluate the broad applicability of FLM@VC across glioma cells, we extended our investigation to the CT‐2A cell line, a less immunogenic and more treatment‐resistant glioma model. First, the biocompatibility of FL, MK‐8931, FLM, FLM@V, and FLM@VC on CT‐2A cells was confirmed via CCK‐8 assay (Figure S12). The data confirmed that all formulations exhibited no significant cytotoxicity within the tested concentration range. Consistent with the findings in GL261 cells, FLM@VC exhibited significant anti‐tumor activity in CT‐2A co‐culture systems. CCK‐8 assays revealed that compared to 100% in the Control group, FL, MK‐8931, FLM, and FLM@VC showed viabilities of 98%, 63%, 65%, and 64%, respectively (Figure S13). The apoptosis rate of CT‐2A cells was significantly higher in the FLM@VC group (22.37%) than in the Control group (8.70%) (Figure S14). Moreover, we examined the phagocytosis of TAMs repolarized from M2 to M1 by exposing M2 TAMs to different treatments and co‐culturing them with CT‐2A cells (Figure S15). Compared to the Control group, M2 TAMs treated with FL showed minimal enhancement (∼1.1‐fold). Significant increases in phagocytosis were observed in the MK‐8931 (∼1.58‐fold), FLM (∼1.72‐fold), and FLM@VC (∼1.62‐fold) groups. Although the magnitude of effects was somewhat attenuated compared to GL261 cells, these results collectively indicated that FLM@VC retains meaningful immunomodulatory and anti‐tumor activity even in less‐responsive glioma models, supporting its potential for broader therapeutic application.

### 2.4 VEGF‐C‐Mediated MLV Remodeling Establishes a Bridge for Efficient Brain Delivery

**FIGURE 4:**
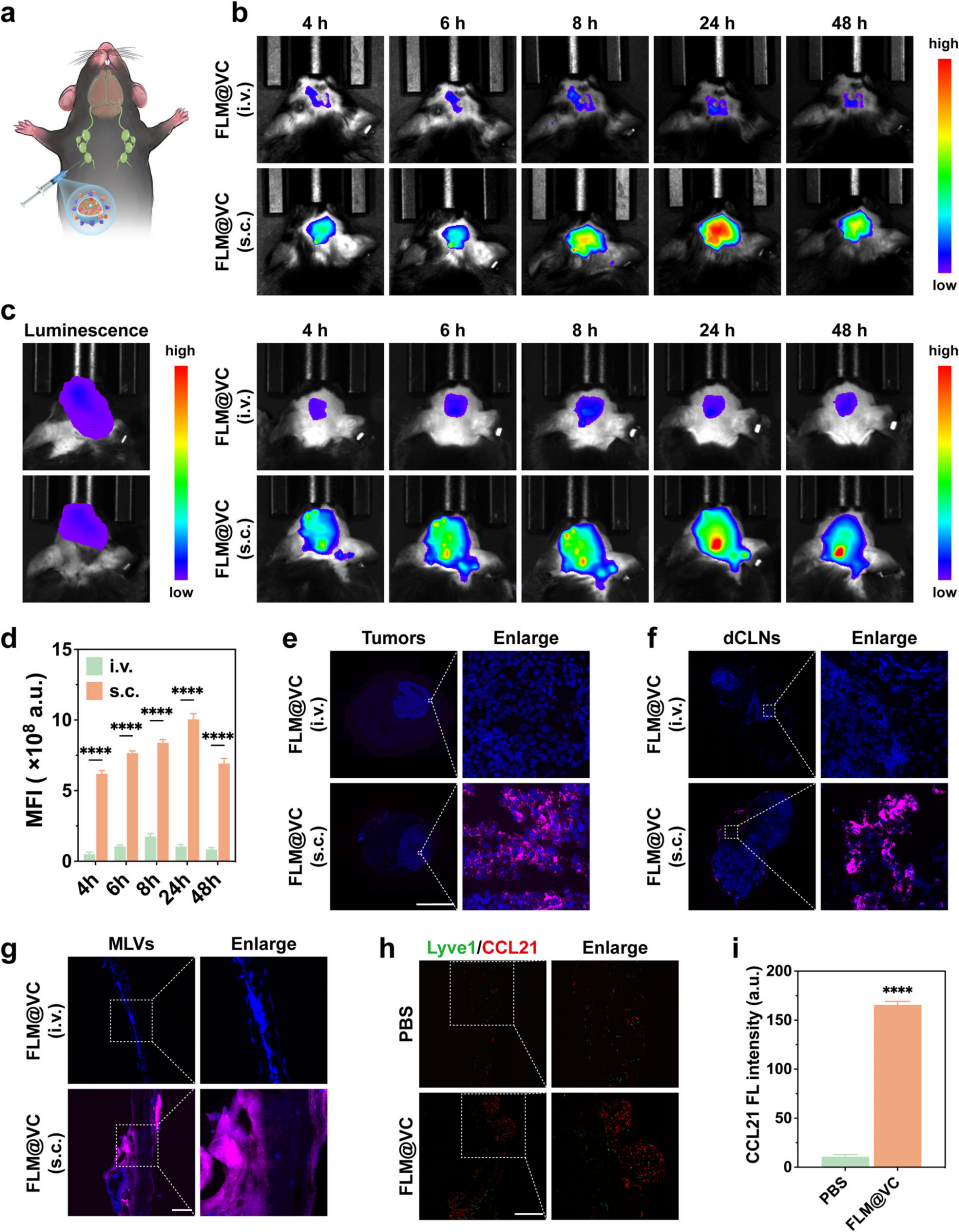
MLVs establish a bridge for efficient brain delivery. (a) Illustration of MLVs‐mediated delivery of FLM@VC into brain tissues. Real‐time fluorescence images of the mice after intravenous (i.v.) injection of Cy5‐labeled FLM@VC and subcutaneous (s.c) injection of Cy5‐labeled FLM@VC in (b) healthy mice and (c) GL261 glioma‐bearing mice. (d) Quantitative analysis of the brain of GL261 glioma‐bearing mice. Data are shown as the mean values ± SD (n = 3). The distribution of Cy5‐labeled FLM@VC in mice at 24 h post s.c. administration is shown in (e) tumors (scale bar: 5 mm), (f) dCLNs (scale bar: 500 µm), and (g) meningeal lymphatic vessels (scale bar: 100 µm). (h–i) Lyve1 and CCL21 staining of MLVs and the corresponding statistical analysis. Scale bar: 500 µm. Data are shown as the mean values ± SD (n = 3). All the statistical significance was analyzed by two‐tailed Student's *t* test. *p < 0.05, **p < 0.01, ***p < 0.001, and ****p < 0.0001, ns, not significant.

Based on the significant inhibitory effect of FLM@VC on tumor cells in vitro, we next explored whether VEGF‐C‐mediated MLV remodeling could serve as a bridge to enhance its delivery to GBM in mice (Figure 4a). First, we explored the transport efficiency of FLM@VC entering the brain through MLVs‐mediated delivery in healthy mice, the Cy5‐labeled FLM@V NPs were intravenously and subcutaneously administered into the mouse at the neck site, respectively. Following subcutaneous (s.c.) injection of FLM@VC, mice maintained a higher fluorescence level from 4 to 48 h compared to those receiving intravenous (i.v.) injection (Figure 4b). Next, we further evaluated this in GL261 glioma‐bearing mice. The results showed significantly higher fluorescence intensity in the brain from Cy5‐labeled FLM@VC after s.c. administration, consistent with that in healthy mice (Figure 4c,d). Specifically, at 24 h, the fluorescence intensity was 11‐fold higher with s.c. injection. To confirm the MLVs‐mediated delivery, we examined the pathway from the initial drainage of FLM@VC at the subcutaneous injection site to the dCLNs, followed by their transport via the MLVs into the brain. The dCLNs, MLVs, and brains were obtained for frozen sections at 48 h post‐administration. Fluorescent scanning images of brain sections showed that subcutaneous injection led to significantly higher accumulation of FLM@VC at the GBM site compared to intravenous administration (Figure 4e), consistent with our in vivo imaging results. As illustrated in Figure 4f,g, following subcutaneous injection, strong fluorescent signals were detected in the dCLNs and the MLVs, whereas almost no detectable fluorescence was observed after intravenous administration. Importantly, no fluorescent signal from the nanoparticles was observed within the BBB delineated by the Claudin‐5 staining, confirming that FLM@VC did not cross the BBB (Figure S16). The data demonstrated that FLM@VC bypassed the BBB entirely via the MLV pathway. The above results indicated that the MLVs established a bridge for more efficient delivery route to the brain than the conventional intravenous injection, which depends on BBB penetration and are often limited by its low permeability and systemic clearance. In addition, it was observed that Cy5‐labeled FLM@VC achieved higher tumor accumulation compared to Cy5‐labeled FLM@V (Figure S17). Decorating the FLM@V with the c(RGDfK) peptides further increased delivery efficiency through MLV route. These findings revealed the sequential targeting capability of FLM@VC from the subcutaneous injection site to dCLNs, subsequently migrated through MLVs, and ultimately achieved enhanced accumulation within the GBM site.

Given the superior efficiency of the MLV route for FLM@VC delivery to the brain, we next sought to elucidate the underlying mechanism by which VEGF‐C facilitates this process. The differences in MLVs between the FLM@VC and Control groups were analyzed. VEGF‐C facilitated MLV expansion, indicated by larger MLV diameters in the FLM@VC group (Figure S18). Notably, CCL21 expression was upregulated in the lymphatic endothelial cells (LECs) of MLVs in the FLM@VC group. (Figure 4h,i). Among LEC‐derived chemokines, CCL21 plays the most critical role in the recruitment of dendritic cells (DCs). Therefore, VEGF‐C could enhance the drainage of tumor antigens, thereby boosting anti‐tumor immune responses through MLV expansion. These results laid a good foundation for the therapeutic effect of FLM@VC NPs.

These findings demonstrated that subcutaneous administration of FLM@VC enabled significantly more efficient delivery to the GBM site than intravenous injection. This enhanced delivery occurred through a sequential targeting pathway where nanoparticles first drained to the dCLNs, then were transported via MLVs to reach the GBM site. Furthermore, decoration with c(RGDfK) peptides augmented delivery efficiency through the MLV route. Meanwhile, VEGF‐C promoted MLV expansion and upregulated CCL21 expression in LECs. These remodeled MLVs served as a conduit not just for FLM@VC, but more importantly, for DCs to traffic to dCLNs. This process is fundamental for priming T cells and initiating a robust peripheral immune response.

### 2.5 Exploration of Anti‐Tumor Effect in Vivo

**FIGURE 5:**
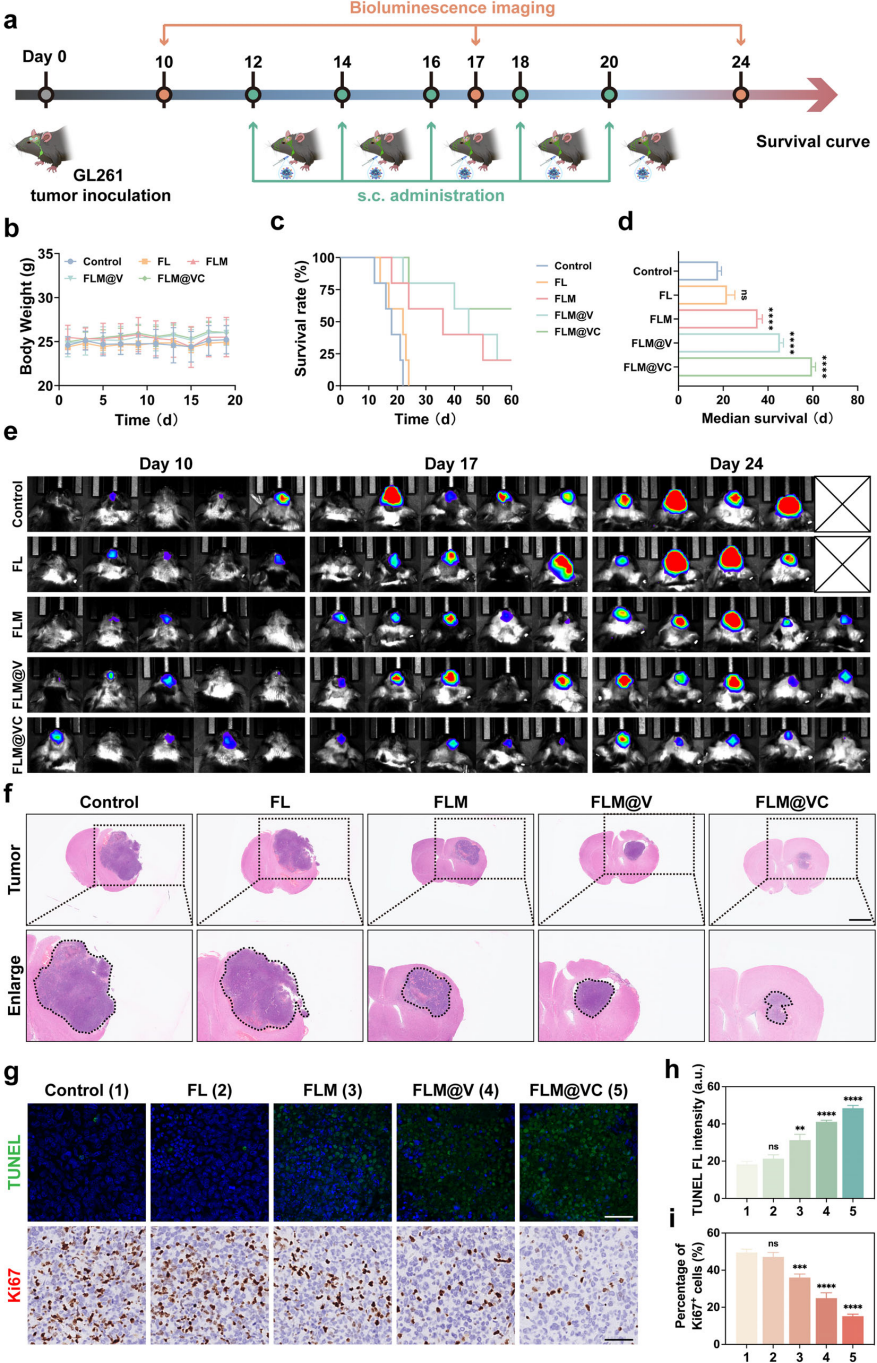
Antitumor evaluation in vivo. (a) Illustration of the experimental design. (b) Change in average body weight of C57BL/6J mice over the observation period. Data are shown as the mean values ± SD (n = 5). (c, d) Survival curves of GL261 glioma‐bearing mice after different treatments (n = 5). (e) Representative bioluminescence images of GL261 glioma‐bearing mice following different treatments (n = 5). (f) H&E‐stained brain sections collected from different groups. Scale bar: 2 mm. (g–i) TUNEL staining and immunohistochemical staining for Ki67 of tumor slices after different treatments and the corresponding statistical analysis. Scale bar: 50 µm. Data are shown as the mean values ± SD (n = 3). All the statistical significance was analyzed by ANOVA with a Tukey post‐hoc test. *p < 0.05, **p < 0.01, ***p < 0.001, and ****p < 0.0001, ns, not significant.

Encouraged by the excellent performance of sequential targeting in GBM delivery, we then evaluated the in vivo anti‐GBM effect of FLM@VC NPs following a brief experimental design (Figure 5a). We developed a GBM model by inoculating mice with GL261 cells to effectively assess the therapeutic efficacy of various treatments. Ten days post‐tumor implantation, the mice were randomized into five groups: 1) Control; 2) FL; 3) FLM; 4) FLM@V; 5) FLM@VC. The body weight of every mouse was also monitored. As shown in Figure 5b, there were no notable changes in body weight in any group during the study, confirming the excellent biocompatibility of FLM@VC NPs. Moreover, we assessed the effectiveness of each treatment group in extending the survival of orthotopic GBM tumor models (Figure 5c,d). The median survival times were 18 days for the Control group, 22 days for FL group, 36 days for FLM group, and 45 days for FLM@V group, with the FLM@VC group showing a significant extension to 60 days. Notably, even after 60 days of detection, 3 out of 5 orthotopic GBM tumor models in the FLM@VC group survived, achieving a 60% survival rate, highlighting the significant advantages of FLM@VC in extending life. Tumor growth was monitored continuously by bioluminescence imaging after tumor implantation and treatment administration. As shown in Figure 5e and Figure S19, tumor volume increased rapidly in the Control and FL groups, resulting in one mouse death in each group on day 24, while tumor growth was partially inhibited in the FLM and FLM@V groups. The growth of orthotopic GBM was significantly suppressed and even abolished when administered with FLM@VC, suggesting that FLM@VC had a significant tumor inhibitory effect.

To explore the therapeutic effect and underlying inhibitory mechanism of FLM@VC, the brains were isolated for hematoxylin and eosin (H&E), TdT mediated dUTP nick end labeling (TUNEL), and immunohistochemical (IHC) staining assay. Figure 5f showed that FLM@VC treatment resulted in minimal tumor size in mice brains compared to other treatments, which aligned with the results of bioluminescence imaging. TUNEL staining showed that compared to Control and FL groups, the TUNEL fluorescence signals were moderately increased in the FLM group, significantly higher in the FLM@V group, while the fluorescence signals were largely increased in the FLM@VC group (Figure 5g,h), suggesting the highest cell death ratio. Moreover, the proportion of Ki67 positive cells was 47.5%, 44.4%, 34.1%, 27.8%, and 15.7% in the Control, FL, FLM, FLM@V, and FLM@VC groups, respectively (Figure 5g,i), suggesting FLM@VC could effectively inhibit the proliferation of GBM cells compared with other treatments and thus improve the survival benefit.

Collectively, these findings confirmed the therapeutic efficacy with favorable survival benefits of FLM@VC NPs in the orthotopic GBM tumor models, rendering FLM@VC a promising nanoplatform for the application of GBM therapy. The mechanism and the alternation of the immunosuppressive TME will be explored in detail in the following parts.

### 2.6 FLM@VC Reprograms Pro‐Tumoral M2 TAMs via BACE1‐STAT3 Inhibition to Activate Intratumoral Immunity

**FIGURE 6:**
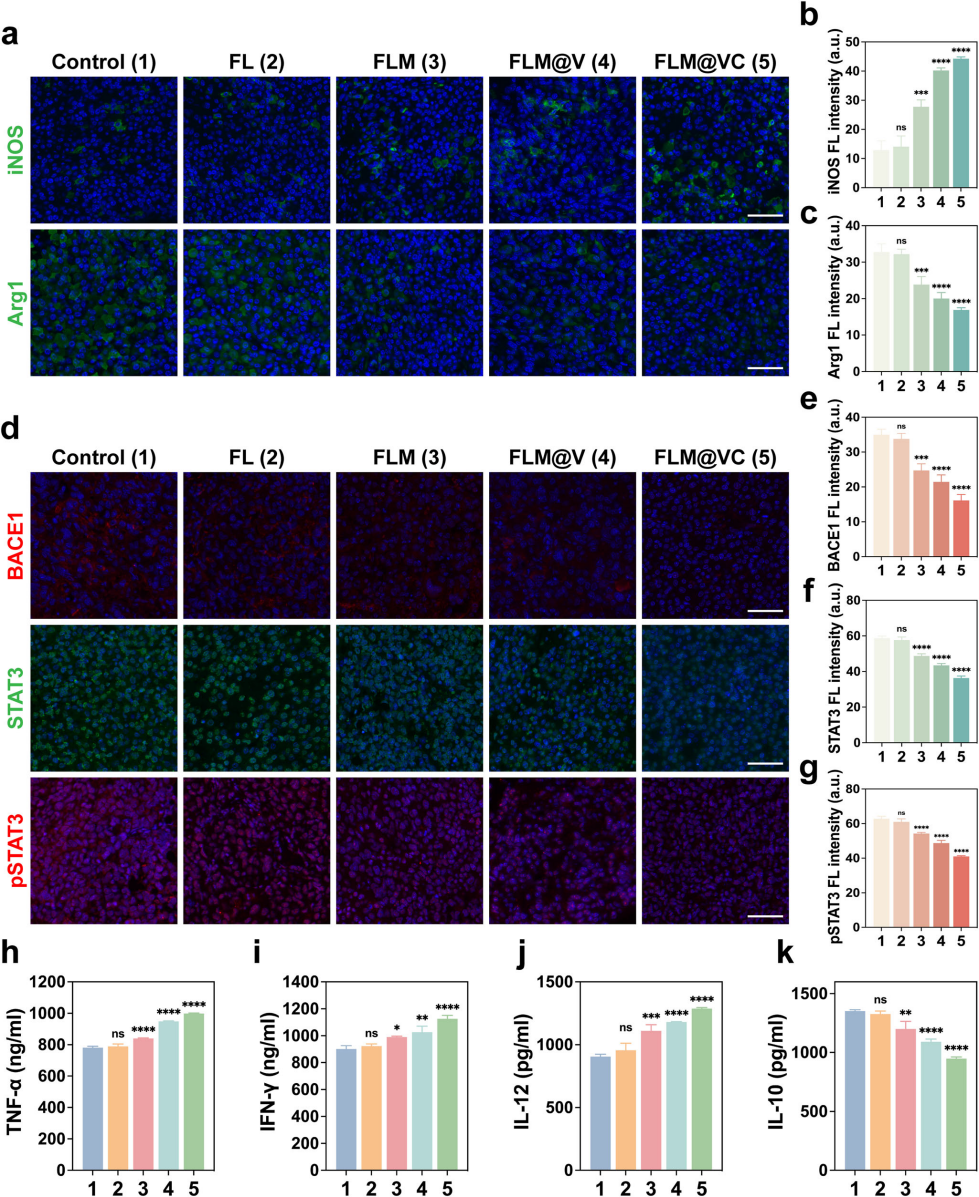
FLM@VC‐mediated TAM reprogramming activates intratumoral immunity. (a) IF staining of iNOS and Arg1 in tumor sections following different treatments. Scale bar: 50 µm. (b, c) Quantitative fluorescence analysis of iNOS and Arg1. Data are shown as the mean values ± SD (n = 3). (d) IF staining of BACE1, STAT3, and pSTAT3 in tumor sections after various treatments. Scale bar: 50 µm. (e–g) Corresponding statistical analysis of BACE1, STAT3, and pSTAT3. Data are shown as the mean values ± SD (n = 3). (h–k) The secretion level of TNF‐α, IFN‐γ, IL‐10, and IL‐12 following different treatments. Data are shown as the mean values ± SD (n = 3). All the statistical significance was analyzed by ANOVA with a Tukey post‐hoc test. *p < 0.05, **p < 0.01, ***p < 0.001, and ****p < 0.0001, ns, not significant.

FLM@VC exhibited satisfactory anti‐GBM effects, we then investigated its effect in reprogramming the TAMs and reshaping the immunosuppressive TME. Immunofluorescence (IF) staining revealed that FLM@VC treatment significantly increased the fluorescence signals of CD86 (Figure S20a,b) and iNOS (Figure 6a,b; Figure S21a) in the GBM tissues. Conversely, it decreased the signals of CD206 (Figure S20a,c) and Arg1 (Figure 6a,c; Figure S21b). These results indicated that FLM@VC significantly reprogrammed the M2 TAMs toward an M1 phenotype. Interestingly, compared to the FLM group, FLM@V and FLM@VC groups significantly increased CD86 and iNOS expression, while CD86 and iNOS levels were reduced, further suggesting that the VEGF‐C modification promoted MLV expansion, enhancing drug delivery to the brain. Moreover, the FLM@VC group exhibited stronger iNOS and CD86 signals and weaker Arg1 and CD206 expression in tumor tissues. This enhanced efficacy can be attributed to the presence of the c(RGDfK) targeting peptide on FLM@VC. This improved localization ensured more efficient release of MK‐8931 within the TME, which was fundamental for the repolarization of TAMs toward an M1 phenotype.

To further unlock the underlying mechanism involved in reprogramming the M2 TAMs toward an M1 phenotype, we continued to examine histological expression of BACE1, STAT3, and pSTAT3 in GL261 tumor tissues via IF staining. As shown in Figure 6d–g, the FLM, FLM@V, and FLM@VC drastically reduced the expression of BACE1, STAT3, and pSTAT3 in comparison with the Control and FL groups, and the FLM@VC group showed the strongest suppression, elucidating that MK‐8931 induced TAM repolarization. In addition, the elevated levels of TNF‐α, IFN‐γ, and IL‐12, along with decreased IL‐10 levels, secreted in FLM@VC‐treated mice indicated that the local immunosuppressive TME was significantly improved (Figure 6h–k).

These results demonstrated that FLM@VC could efficiently deliver MK‐8931 to the GBM site and thus effectively reprogram M2 TAMs to M1 TAMs through BACE1‐STAT3 signaling. This repolarization greatly reversed the immunosuppressive TME, fostering an immunostimulatory niche, which was beneficial for subsequent CD8^+^ T cell infiltration and killing.

### 2.7 Evaluation of MLVs‐Bridged Intracranial‐Peripheral Dual Immune Modulation in Vivo

**FIGURE 7:**
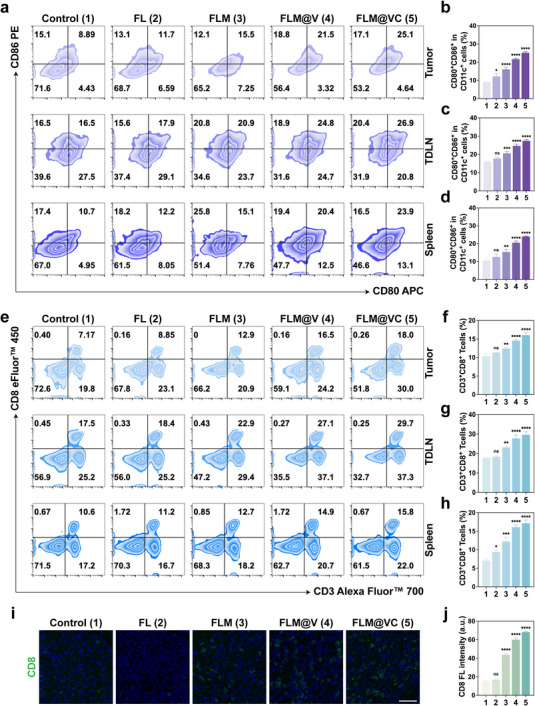
Dual immune modulation assessment in vivo. (a) Representative FCM results of mDCs within tumors, TDLNs, and spleens following different treatments. (b–d) Corresponding statistical analysis of DC maturation within tumors, TDLNs, and spleens following different treatments. Data are shown as the mean values ± SD (n = 3). (e) Representative FCM results of CD3^+^CD8^+^ T cells within tumors, TDLNs, and spleens following different treatments. (f‐h) Corresponding statistical analysis of CD3^+^CD8^+^ T cells proportion within tumors, TDLNs, and spleens following different treatments. Data are shown as the mean values ± SD (n = 3). (i) IF staining of CD8^+^ T cells in tumor sections following various treatments. Scale bar: 50 µm. (j) Corresponding statistical analysis of CD8^+^ T cells. Data are shown as the mean values ± SD (n = 3). All the statistical significance was analyzed by ANOVA with a Tukey post‐hoc test. *p < 0.05, **p < 0.01, ***p < 0.001, and ****p < 0.0001, ns, not significant.

The polarization state of TAMs profoundly influenced the immune milieu to activate intratumoral immunity. Building upon this, we next explored the maturation of DCs and the CD8^+^ T cell activation within this context. Furthermore, we demonstrated how FLM@VC empowered MLVs‐bridged intracranial‐peripheral dual immune modulation. As professional antigen‐presenting cells (APCs), mature dendritic cells (mDCs), play a pivotal role in priming and activating CD8^+^ T cell responses. Compared to the Control group (∼8.89%), the proportion of mDCs showed minimal change in the FL NPs group (∼11.7%), a slight increase in the FLM group (∼15.5%), and remarkable increases in the FLM@V (∼21.5%) and FLM@VC (∼25.1%) groups, particularly in the latter group, with a nearly 2.8‐fold increase (Figure 7a,b). The DC maturation in tumor draining lymph nodes (TDLNs) and the spleen was evaluated by FCM to further assess the immunostimulatory effect of FLM@VC against GBM. As illustrated in Figure 7a,c, the proportion of mDCs in TDLNs increased following treatment with FLM, FLM@V, and FLM@VC, compared to the Control and FL groups, with the FLM@VC group exhibiting the highest proportion. These results were likely due to three key factors: 1) TAMs reprogramming improved antigen presentation and promoted DC maturation; 2) VEGF‐C‐induced lymphangiogenesis enhanced DC trafficking; 3) efficient targeting of FLM@VC mediated by VEGF‐C and c(RGDfK) peptides enhanced drug delivery. The proportion of mDCs in the spleen was consistent with the trends observed in both the tumor and TDLNs (Figure 7a, d), confirming the efficacy of FLM@VC in promoting DC maturation.

To further validate that VEGF‐C‐mediated MLV expansion enhances DC trafficking to dCLNs, we performed FCM analysis of dCLNs harvested from treated mice (Figure S22). The results demonstrated a significant increase in the proportion of mDCs in the FLM@V and FLM@VC groups compared to other groups. These findings confirmed that VEGF‐C not only promoted MLV structural expansion but also functionally enhanced DC trafficking.

Since FLM@VC showed a strong capability to promote DC maturation, we further investigated its ability to activate CD8^+^ T cells. Excitingly, the FLM@VC, FLM@V, and FLM groups all showed increased frequency of tumor‐infiltrating CD3^+^CD8^+^ T cells compared to the Control and FL groups, with the most remarkable increase observed in the FLM@VC group (Figure 7e,f, i, j). FLM@VC treatment induced a systemic immune activation, as evidenced by elevated CD3^+^CD8^+^ T cell percentages in both TDLNs and the spleen, where it achieved the highest levels among all groups (Figure 7e, h, g). To further assess the functional state of infiltrating CD8^+^ T cells, we performed IF staining for T cell activation and T cell exhaustion. As shown in Figure S23, FLM@VC treatment increased the proportion of CD8^+^CD69^+^ T cells while maintaining a lower fraction of CD8^+^TIM3^+^ T cells within tumor tissues, indicating that the majority of CD8^+^ T cells were in an activated state. These results confirmed that the FLM, FLM@V, or the FLM@VC NPs stimulated varying levels of immune responses intratumorally and systemically. To be specific, FLM group was found to recruit more CD3^+^CD8^+^ T cells than the Control and FL groups, which confirmed the ability of MK‐8931 to repolarize TAMs and stimulate the immune landscape in the TME. Notably, the observed increases in CD8^+^CD69^+^ T cells within the FLM@V and FLM@VC groups can be attributed to VEGF‐C‐mediated enhancement of DC trafficking and subsequent CD8^+^ T cell activation. Moreover, the FLM@VC exhibited the strongest immunostimulatory effect, indicating that the combination of MK‐8931 and VEGF‐C enhanced the anti‐tumor immune response, potentially leading to an increased survival rate of mice in the FLM@VC group.

Taken together, these findings demonstrated the enhanced ability of FLM@VC to activate intracranial‐peripheral dual immune responses. On the one hand, FLM@VC delivered more drugs to the GBM site, resulting in enhanced therapeutic efficacy. On the other hand, VEGF‐C promoted DC trafficking and maturation, facilitating tumor antigen presentation and the subsequent activation of CD8^+^ T cells, leading to an enhanced anti‐GBM immune response.

### 2.8 Biosafety and Biodistribution of FLM@VC

An ideal biomimetic nanoparticle needs to have not only suitable physical and chemical properties but also high biocompatibility and minimal toxicity. The biological safety of FLM@VC was evaluated by monitoring hematological and biochemical parameters on days 0, 1, 3, 7, 14, and 21 post‐treatment. On day 21, all mice were sacrificed to obtain whole blood, serum, and major organs for hematological analysis, liver and kidney function assessment, and histological examination. As shown in Figure S24, the levels of WBC, RBC, HGB, PLT, Gran, MCV, ALT, AST, BUN, CREA, CK, and LDH showed no significant differences, suggesting no hematological, hepatic, or renal toxicity. In addition, H&E staining revealed no significant histopathological changes in major organs (heart, liver, spleen, lung, and kidney) (Figure S25). Furthermore, FLM@VC caused no significant hemolysis even at a higher concentration (up to 200 µg mL^−1^), demonstrating the good hemocompatibility of FLM@VC NPs (Figure S26).

For biodistribution analysis, the tumors and major organs (heart, liver, spleen, lung, and kidney) were dissected at various time points (4, 6, 8, 24, and 48 h) post‐administration and monitored. The ex vivo fluorescence imaging showed that the kidney displayed the strongest fluoresce intensity, followed by the tumor, liver, while the spleen, heart, and lung exhibited negligible fluorescence (Figures S27 and S28). The reason why the FLM@VC accumulated the most in the kidney might be due to its role as the primary filtration and excretory organ, coupled with extensive blood perfusion. Following cervical lymph node injection and entry into systemic circulation, drug components small enough for glomerular filtration or released fluorescent metabolites are rapidly concentrated within the renal tubules during urine formation, leading to the high observed fluorescence intensity.

These data effectively evidenced that FLM@VC possessed excellent biocompatibility in vivo, with no significant hematological, hepatic, renal, or histopathological toxicity observed, and exhibited a favorable biodistribution profile characterized by significant accumulation in the target tumor tissue, making it a promising alternative for future clinical application.

Although MK‐8931 encountered clinical challenges in Alzheimer's disease trials due to adverse events and insufficient efficacy, its application within the FLM@VC may present a distinct safety profile. FLM@VC utilized the MLVs as a targeted delivery route to the brain, concentrating the MK‐8931 within the TME. This localized delivery not only enhanced the reprogramming of TAMs from pro‐tumoral M2 to anti‐tumoral M1 TAMs, but also significantly reduced off‐target exposure and potential systemic toxicity. We will actively explore these safety advantages in our subsequent studies to further advance its translational potential.

## 3 Conclusion

In summary, we successfully constructed a novel metal‐supramolecular delivery system (FLM@VC) using a self‐assembly approach. This system exhibited favorable biocompatibility, TME responsiveness, and leveraged the MLVs as a bridge to achieve intracranial‐peripheral dual immune modulation for reversing immunosuppression in GBM. Notably, we utilized the MLV pathway for the effective transport of FLM@VC into the brain, overcoming the limitations of conventional CNS delivery methods. This system synergistically integrates two immunomodulatory strategies: intracranial reprogramming of TAMs from pro‐tumoral M2 to the anti‐tumoral M1 phenotypes via BACE1‐STAT3 inhibition, and peripheral remodeling of MLVs to facilitate antigen presentation, activating CD8^+^T cells and enhancing anti‐GBM immune responses. As a result, FLM@VC treatment transformed “cold” GBM into an immunologically “hot” tumors, resulting in favorable tumor inhibition and prolonged survival benefits. This work highlights the immense potential of orchestrated immunomodulation that simultaneously targets both the peripheral lymphatic system and the intracranial TME. Beyond GBM, this MLVs‐guided delivery platform offers a versatile and promising strategy for the treatment of a broad spectrum of brain diseases.

## 4 Experimental Section

### 4.1 In Vitro Investigation of Macrophage Polarization

RAW264.7 cells were seeded in 6‐well plates (2 × 10^5^ cells per well) and cultured overnight. Next, cells were exposed to IL‐4 (40 ng/mL) for 48 h to induce M2 polarization. Cells were then treated with PBS, FL, MK‐8931, FLM, and FLM@VC. Twelve hours post co‐incubation, the cells were stained with PE anti‐CD86 antibody and APC anti‐CD206 antibody for FCM analysis, following the manufacturer's suggestions.

### 4.2 Macrophage Polarization, DC Maturation, and T Cell Activation In Vivo

To study the mechanism underlying FLM@VC‐induced macrophage polarization, tumor tissues of each group were obtained for IF staining to assess the expression of CD86, CD206, Arg1, iNOS, BACE1, STAT3, and pSTAT3. Brain, tumor‐draining lymph nodes (TDLNs), and spleen tissues were cut into small pieces, and digested with a solution containing collagenase D (1 mg/mL), dispase (1 mg/mL), and DNase I (0.5 mg/mL) in DMEM at 37°C for 30 min. Cells were blocked with CD16/CD32, then stained with Alexa Fluor 700 anti‐mouse CD3, eFluor 450 anti‐mouse CD8, FITC anti‐mouse CD11c, APC anti‐mouse CD80, PE anti‐mouse CD86. Finally, samples were detected by FCM. To assess the anti‐tumor immune response, brain tissues were stained with CD8, CD69, and TIM3. Cytokine levels (IFN‐γ, TNF‐α, IL‐12, and IL‐10) were quantified using ELISA according to the manufacturer's instructions.

### 4.3 Animals Experiments

C57BL/6J mice (6–8 weeks, male) were purchased by Enswell Biotechnology Ltd (Chongqing, China). All animal experiments were approved by the Ethics Committee of Chongqing Medical University, Approval No. IACUC‐SAHCQMU‐2024‐00109.

### 4.4 Human Tumor Specimens

The tumor tissues of GBM patients were sourced from the Department of Neurosurgery at the Second Affiliated Hospital of Chongqing Medical University. All specimens were obtained following written informed consent and in accordance and the research protocol approved by the Ethics Committee of the Second Affiliated Hospital of Chongqing Medical University (Approved No. YLS 2025–301).

### 4.5 Statistical Analysis

Each experimental condition was independently repeated at least three times. All quantitative data are shown as the mean ± standard deviation (SD). Statistical analysis was performed using GraphPad 9. The Student's *t*‐test was conducted to assess the significant differences between the two groups. A one‐way analysis of variance (ANOVA) was conducted for multiple group comparisons, followed by Tukey's post‐hoc test. Significance was determined at *p*‐values of *p < 0.05, **p < 0.01, ***p < 0.001, and ****p < 0.0001.

A more detailed experimental section is available from the Supporting Information.

## Author Contributions

C.Z. is the first author, and Z.X. and X.X. are the parallel first author. They contributed equally to this study, including conceptualization, methodology, investigation, validation, data analysis. Specifically, C.Z. is responsible for processing raw data and writing the manuscript, while X.X. and Z.X. are responsible for reviewing and revising the draft. Z.Z., R.L., and P.R. contributed partially to the methodology part. Y.L., Q.W., and X.L. contributed partially to the study administration part. G.L., X.H. and Y.L. are the corresponding author and contributed mainly to the Funding part and paper revision.

## Conflicts of Interest

The authors declare no conflicts of interest.

## Supporting information




**Supporting File**: advs74281‐sup‐0001‐SuppMat.pdf.

## Data Availability

The data that support the findings of this study are available from the corresponding author upon reasonable request.
