# Supplementary material for: Metal‐Supramolecular Drug Delivery System Empowered Meningeal Lymphatic Vessels‐Bridged Intracranial‐Peripheral Dual Immune Modulation for Reversing Glioblastoma Immune Suppression
Source: Adv Sci (Weinh). 2026 Feb 8;13(22):e22604. doi: 10.1002/advs.202522604 (PMC13088265; doi:10.1002/advs.202522604)
Supplement: Supplementary file 1 — Supporting File: advs74281‐sup‐0001‐SuppMat.pdf. [file ADVS-13-e22604-s001.pdf]

Metal-Supramolecular Drug Delivery System Empowered Meningeal Lymphatic Vessels-Bridged Intracranial-Peripheral Dual Immune Modulation for Reversing Glioblastoma Immune Suppression

Chenxi Zhang, Zhongsheng Xu, Xiaowen Xu, Zening Zhang, Ranran Luo, Pengchen Ren, Yingying Luo, Qiuchi Wu, Xinyu Liu, Guodong Liu, Xiaojing He*, Yun Liu**

C. Zhang, Z. Xu, Z. Zhang, R. Luo, P. Ren, Y. Luo, Q. Wu, X. Liu, X. He, Y. Liu
Department of Radiology
The Second Affiliated Hospital of Chongqing Medical University
Chongqing 400010, China
E-mail: yunliu@cqmu.edu.cn (Yun Liu); he_xiaojing@hospital.cqmu.edu.cn (Xiaojing He)

X. Xu, G. Liu
Department of Neurosurgery
The Second Affiliated Hospital of Chongqing Medical University
Chongqing 400016, China
E-mail: 304678@hospital.cqmu.edu.cn (Guodong Liu)

Experimental section

Chemical and materials

BSA (P1628334) and α-Lipoic acid (13428B) were obtained from Admas-beta (Shanghai, CHN). Iron(Ⅲ) chloride hexahydrate ($FeCl_3·6H_2O$) (G10101B) and Sodium hydroxide (NaOH) (G19852G) were purchased from General-reagent (Shanghai, CHN). MK-8931 (1286770-55-5) was acquired from Aladdin (Shanghai, China). Cyclo (Arg-Gly-Asp-d-Phe-Lys) (507852) was obtained from GL Biochem (Shanghai, CHN). Vascular Endothelial Growth Factor C Human Recombinant

(VEGF-C Human) (GP20150) was purchased from GLpBio (USA). The Dulbecco's modified Eagle's medium (DMEM) (PYG0073) was purchased from Boster (Wuhan, China). Paraformaldehyde (P0099), streptomycin-penicillin (C0222), trypsin (C0201), DAPI staining agent (C1002), Cell Counting Kit-8 (CCK8) (C0037), and Calcein/PI Cytotoxicity Assay Kit were purchased from Beyotime (Shanghai, China). Sulfo-Cyanine5 was aquired from MCE (New Jersey, USA). Fetal bovine serum (FBS, 10100147) was obtained from Gibco (Thermo Fisher Scientific, Shanghai, China). Mouse IL-4 protein (PRP2117) was acquired from Abbkine (Wuhan, China). Annexin V-FITC/PI Apoptosis Kit (E-CK-A211) was purchased from Elascience Biotechnology (Wuhan, China). Calcein-AM/PI Double Staing Kit (C542) was bought from Dojindo Laboratories (Kumamoto, Japan). The enzyme-linked immunosorbent assays (ELISAs), including Mouse IL-6 (AF2163-A), IL-10 (AF2176-A), IL-12 (AF30084-A), TNF-α (AF2132-A), IFN-γ (AF2182-A) kits were purchased from AiFang (Hunan, China). BACE1 (D10E5) Rabbit mAb (5606T), Stat3 (D3Z2G) Rabbit mAb (12640T), and Phospho-Stat3 (Tyr705) (D3A7) XP® Rabbit mAb (9145T) were acquired from CST (USA). PE anti-mouse CD80, APC anti-mouse CD80, PE anti-mouse CD86, FITC anti-mouse CD11c, APC anti-mouse CD206, eFluor™ 450 anti-mouse CD8, and Alexa Fluor™ 700 anti-mouse CD3 were purchased from Thermo Fisher (Invitrogen, USA). All the chemicals were used as purchased without further purification.

Synthesis and characterization of FL, FLM, FLM@V and FLM@VC

Preparation of FL NPs. First, 10 mL BSA was mixed with 1 ml $FeCl_3·6H_2O$, followed by incubation at room temperature for 30 min. A precipitation agent composed of 40 mM NaOH and 40 mM lactic acid (LA) in 1:1 *v/v* ratio was freshly prepared, and 1 mL of this mixture was added dropwise to the BSA/$FeCl_3$ system. The reaction proceeded for 30 min at 25℃ with continuous stirring (800 rpm). The resultant colloid was purified by dual-cycle centrifugation (10,000 rpm, 10 min) to obtain FL NPs.

Preparation of FLM NPs. 10 mL BSA solution and 1 mL $FeCl_3·6H_2O$ (0.1 M) were

magnetically stirred (800 rpm, 25℃) for 30 min, after which 100 μL MK-8931 (10 mg/mL in DMSO) was incorporated under continued stirring for 30 min. A 1 mL aliquot of NaOH/LA mixture (40 mM each, 1:1 *v*/*v*) was then added dropwise, allowing 30 min reaction. Purification was performed identically to the FL NPs protocol.

Preparation of FLM@V NPs. FLM@V NPs were obtained by first preparing FLM NPs as described above, followed by the dropwise addition of VEGF-C (50 μg) and incubation under agitation for 30 min. The resulting nanoparticles were purified by centrifugation (10,000 rpm, 10 min, twice).

Preparation of FLM@VC NPs. FLM@VC NPs were prepared similarly to FLM NPs, with VEGF-C (50 μg) and c(RGDfK) (20 μg) sequentially added dropwise with 10-min intervals after the NaOH/LA mixture incorporation. The final product was purified via centrifugation (10,000 rpm, 10 min, twice).

Lastly, Cy5-labeled FLM@V and Cy5-labeled FLM@VC were prepared by mixing the respective membranes with Cy5-NHS, stirring at 300 rpm for 2 h at 25℃.

The hydrodynamic diameters and zeta potentials of the FLM@VC were analyzed by a surface zeta potential and particle size analyzer at room temperature (Zetasizer lab, Malvern, Britain). The morphology of FLM@VC was observed using transmission electron microscopy (HT7700, Hitachi, Japan). XPS was carried out with an ESCALAB 250Xi spectrometer (Thermo escalab 250XI) equipped with an achromatic Al-Kα X-ray source. FT-IR (Nicolet iS5, Thermo Fisher Scientific, USA) was performed to determine the infrared absorption spectrum of MK-8931 in FLM@VC.

Cell Culture

RAW264.7 cells, GL261 cells, and HK-2 cells were generously donated by the central laboratory of the Second Affiliated Hospital of Chongqing Medical University. CT-2A cells were generously donated by the Institute for Brain Science and Disease of Chongqing Medical University. RAW264.7 cells, GL261 cells, and CT-2A cells were cultured in DMEM containing 10% fetal FBS, penicillin (100 U/mL) and

streptomycin (100 mg/mL) under a humidified atmosphere of 5% CO_2 at 37℃. HK-2 cells were cultured in MEM with 10% FBS, penicillin (100 U/mL) and streptomycin (100 mg/mL) under a humidified atmosphere of 5% CO_2 at 37℃. M2 TAMs were obtained by culturing RAW264.7 cells with interleukin 4 (IL-4) (40 ng/mL) overnight.

Cytotoxicity of FL, FLM, FLM@V and FLM@VC

To assess biocompatibility, M2 TAMs cells, GL261 cells cells and HK-2 cells were treated with different concentrations of FL, FLM, FLM@V and FLM@VC (25, 50, 100, 200 μg/mL) for 24 hours, and cell viability was measured using the CCK-8 assay.

The intracellular uptake of Cy5-labeled FLM@VC

The phagocytosis of Cy5-labeled FLM@V and Cy5-labeled FLM@VC was explored in GL261 cells using confocal laser scanning microscopy (CLSM) (Olympus, Tokyo, Japan) and flow cytometry (FCM) (FACS Vantage SE, Becton Dickinson, San Jose, CA, USA). For CLSM, GL261 cells (1×10^5 cells per well) were separately cultured in con-focal dishes overnight. Then the cells were incubated with Cy5-labeled FLM@V and Cy5-labeled FLM@VC for 1 h, 2 h, 4 h and 6 h. After that, the cells were fixed with 4% paraformaldehyde and imaged on a CLSM. For FCM analysis, and cells were seeded in 12-well plate (2×10^5 cells per well). After being incubated with Cy5-labeled FLM@V and Cy5-labeled FLM@VC for 1 h, 2 h, 4 h and 6 h, cells were harvested and resuspended in PBS for FCM analysis.

***In vitro* investigation of macrophage polarization**

RAW264.7 cells were seeded at 2×10^5 cells per well in 6-well plates and cultured overnight. Then, cells were treated with IL-4 (40 ng/mL) for 48 hours to induce M2 polarization. Cells were then treated with PBS, FL, MK-8931, FLM, and FLM@VC. Twelve hours post co-incubation, the cells were stained with PE anti-CD86 antibody and APC anti-CD206 antibody for FCM analysis, following the manufacturer's suggestions.

mRNA Sequencing and Analysis

RAW264.7 cells were seeded in 6-well plates (2×10^5 cells per well), and cultured.

After the treatments, RNA was extracted from the samples using TRIzol reagent (Invitrogen). Each experiment was conducted in triplicate, and RNA sequencing was performed by Bioprofile (Shanghai, China).

Western Blotting

RAW264.7 cells were harvested and subjected to protein extraction. Subsequently, 20 μg of each sample was separated using 10% SDS-PAGE. The proteins were then transferred onto a 0.22 μm polyvinylidene difluoride membrane (PVDF, Millipore, USA). The antibodies employed for immunoblotting were detailed in materials section. A BeyoECL Plus chemiluminescence kit (P0018S, Beyotime) was applied to observe the protein bands.

***In vitro* antitumor effect in a coculture system**

The antitumor effects of macrophage polarization were evaluated using a co-culture transwell system (6-well plate, 0.4 μm-sized microporous membrane). RAW264.7 cells (1×10^5 cells per well) were seeded in the upper chamber of the transwell, while GL261 cells (2×10^5 cells per well) was seeded on the bottom chamber for growth overnight. Following cell attachment, RAW264.7 cells were stimulated to become M2 TAMs. Subsequently, the control group was replaced with pure DMEM medium without fetal bovine serum, while the experimental group was replaced with DMEM medium containing FL, MK-8931, FLM, and FLM@VC. Culture supernatants were collected for cytokine detection (IL-12, IL-10, TNF-α, IFN-γ), and the cells were obtained for further analysis. For cell apoptosis detection, the GL261 cells were washed with PBS, trypsinized, and collected. The cells were then suspended in PBS, and stained with Annexin V-FITC and PI according to manufacturer's instructions. Finally, the cells were washed and resuspended in 300 μL PBS and detected by FCM analysis. Cell viability was assessed by CCK8 and live/dead cell staining.

Phagocytosis of tumor cells by repolarized M2 TAMs

M2 TAMs were treated with PBS, FL, MK-8931, FLM and FLM@VC for 24 h. The treated M2 TAMs labeled with DiR were co-cultured with DiO-labeled GL261 cells for 3 h at 37 ℃ and then photographed and recorded by immunofluorescence using a confocal microscope. And the phagocytosis of tumor cells by macrophages was

detected flow cytometry.

Animals and Tumor Model Establishment

C57BL/6J mice (6-8 weeks, male) were purchased by Enswell Biotechnology Ltd (Chongqing, China). All animal experiments were approved by the Ethics Committee of Chongqing Medical University, Approval No. IACUC-SAHCQMU-2024-00109. The permit number for the animal experiments is Research Ethics Review No. 223 (2023). To establish the orthotopic GBM model, GL261 cells (3×10^5/μL, 5 μL) were inoculated into the right striatum (lateral: 1.8 mm, longitudinal: 0.6 mm, depth: 2.5 mm).

Bioinformatic analysis of GBM patients

The effect of TAMs expression level of GBM patients was analyzed based on publicly available data (GSE138794) from GEO datasets (https://www.ncbi.nlm.nih.gov/geo).

***In vivo* fluorescence imaging**

Cy5-labeled FLM@VC was subcutaneously injected into the MLVs of mice. Mice with intravenous injection of Cy5-labeled FLM@VC was taken as control. The fluorescence images were captured by an *in vivo* fluorescence imaging system (AniView100 pro, China).

Evaluation of antitumor effect *in vivo*

Histological analysis, survival monitoring, body weight tracking and bioluminescence imaging (BLI) were conducted on specific groups of mice. Three mice per group were euthanized on day 25 for brain tumor histological analysis using hematoxylin and eosin (H&E) and terminal deoxynucleotidyl transferase dUTP nick end labeling (TUNEL) staining.

Macrophage polarization, DC maturation and T cell activation *in vivo*

To study the mechanism underlying FLM@VC-induced macrophage polarization, tumor tissues of each group were obtained for IF staining to assess the expression of CD86, CD206, Arg1, iNOS, BACE1, STAT3, and pSTAT3. Brain, tumor-draining lymph nodes (TDLNs) and spleen tissues were cut into small pieces, and digested with a solution containing collagenase D (1 mg/mL), dispase (1 mg/mL), and DNase I

(0.5 mg/mL) in DMEM at 37 ℃ for 30 minutes. Cells were blocked with CD16/CD32, then stained with Alexa Fluor™ 700 anti-mouse CD3, eFluor™ 450 anti-mouse CD8, FITC anti-mouse CD11c, APC anti-mouse CD80, PE anti-mouse CD86. Finally, samples were detected by FCM. To assess the anti-tumor immune response, brain tissues were stained with CD8, CD69, and TIM3. Cytokine levels (IFN-γ, TNF-α, IL-12, and IL-10) were quantified using ELISA according to the manufacturer's instructions.

Biosafety assay of FLM@VC *in vivo*

The biosafety of the FLM@VC *in vivo* was evaluated on C57 BL6/J mice (male, 6-8 weeks). The mice were randomized into six groups (Control group and 1st, 3th, 7th, 14th, 21th day group after injection of FLM@VC, n = 3 each group). On the 21th day, all mice were sacrificed to obtain blood samples and major organs (heart, liver, spleen, lung, and kidney).

Statistical analysis

Each experimental condition was independently repeated at least three times. All quantitative data are shown as the mean ± standard deviation (SD). Statistical analysis was performed using GraphPad 9. The Student's t-test was conducted to assess the significant differences between the two groups. A one-way analysis of variance (ANOVA) was conducted for multiple group comparisons, followed by Tukey's post-hoc test. Significance was determined at p-values of *P<0.05, **P<0.01, ***P<0.001, and ****P<0.0001.

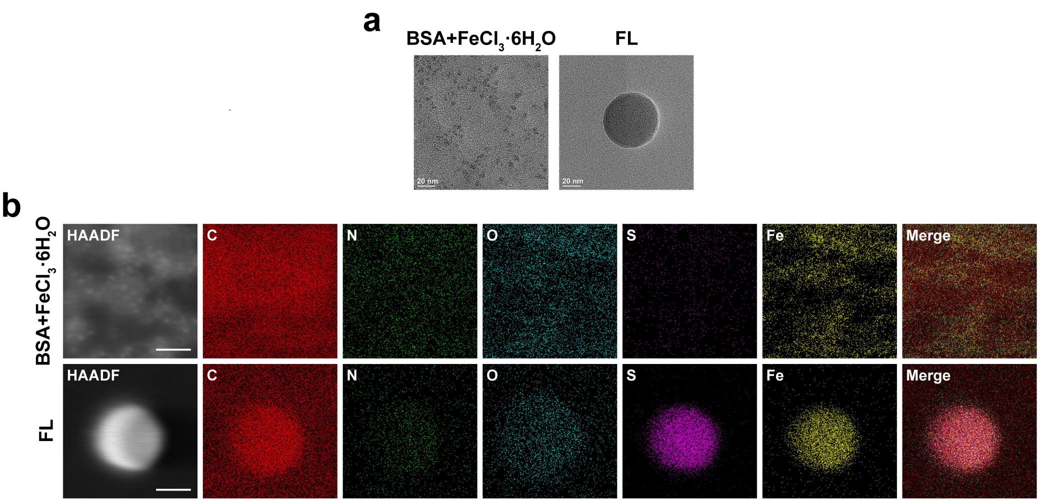

Figure S1. (a) HRTEM of BSA+$FeCl_3·6H_2O$ and FL. Scale bar: 50 nm. (b) Elemental mapping image of BSA+$FeCl_3·6H_2O$ and FL. Scale bar: 50 nm.

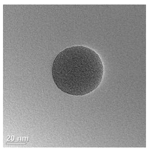

Figure S2. HRTEM of FLM@VC. Scale bar: 20 nm.

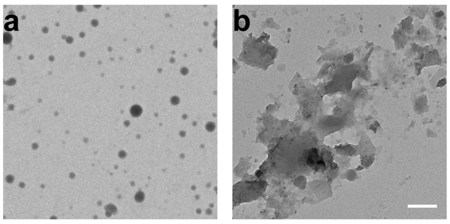

Figure S3. TEM images of (a) FLM@VC and (b) FLM@VC under 5 mM GSH. Scale bar: 200 nm.

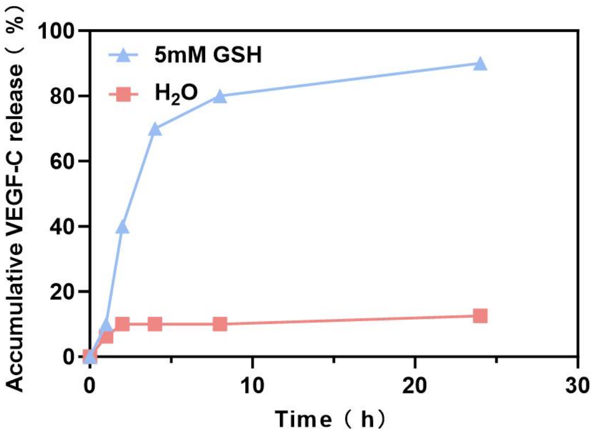

Figure S4. Accumulative release rate of VEGF-C under H_2O and 5 mM GSH.

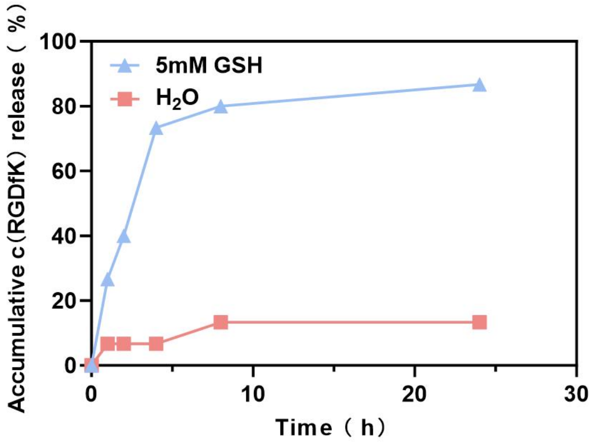

Figure S5. Accumulative release rate of c(RGDfK) under H_2O and 5 mM GSH.

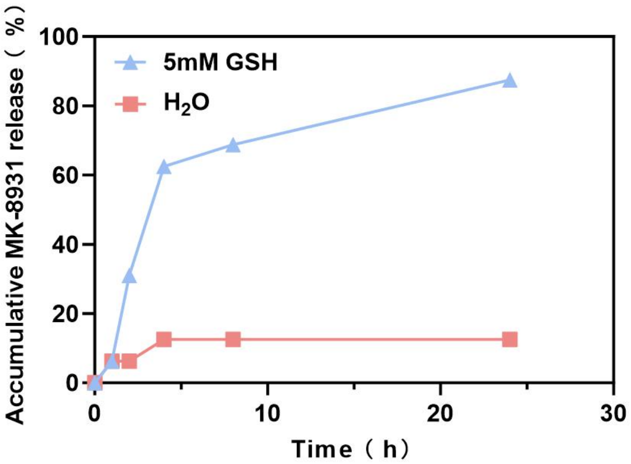

Figure S6. Accumulative release rate of MK-8931 under H_2O and 5 mM GSH.

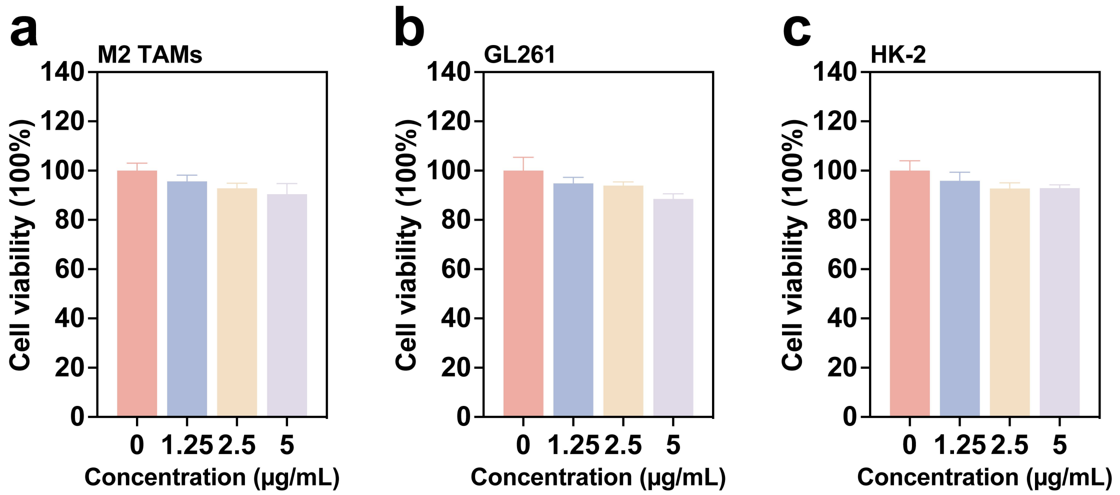

Figure S7. Cell viability of M2 TAMs, GL261 cells, and HK-2 cells after co-incubation with MK-8931. Data are shown as the mean values ± SD (n = 3).

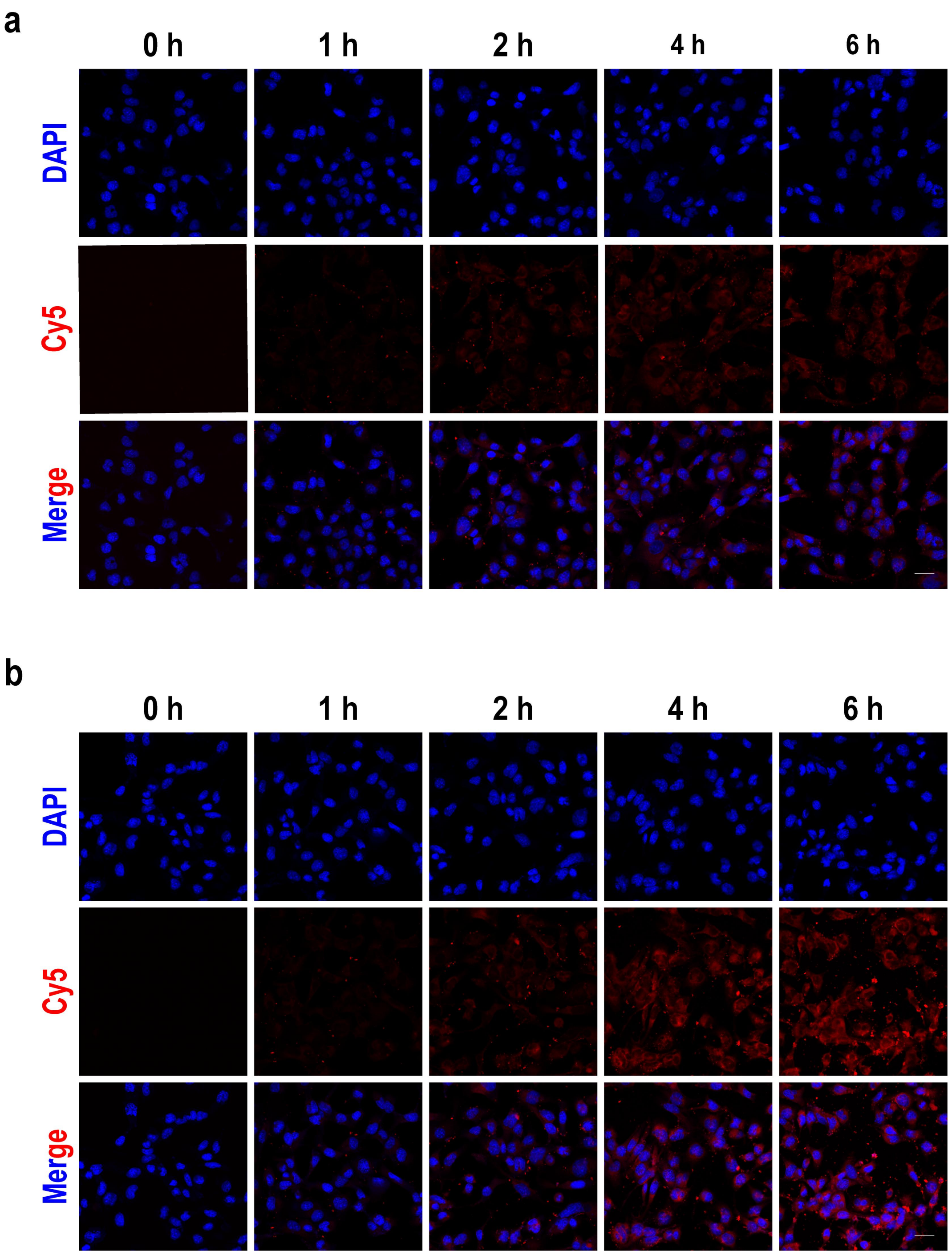

Figure S8. Intracellular uptake of (a) Cy5-labeled FLM@V and (b) FLM@VC under CLSM. Scale bar: 25 μm.

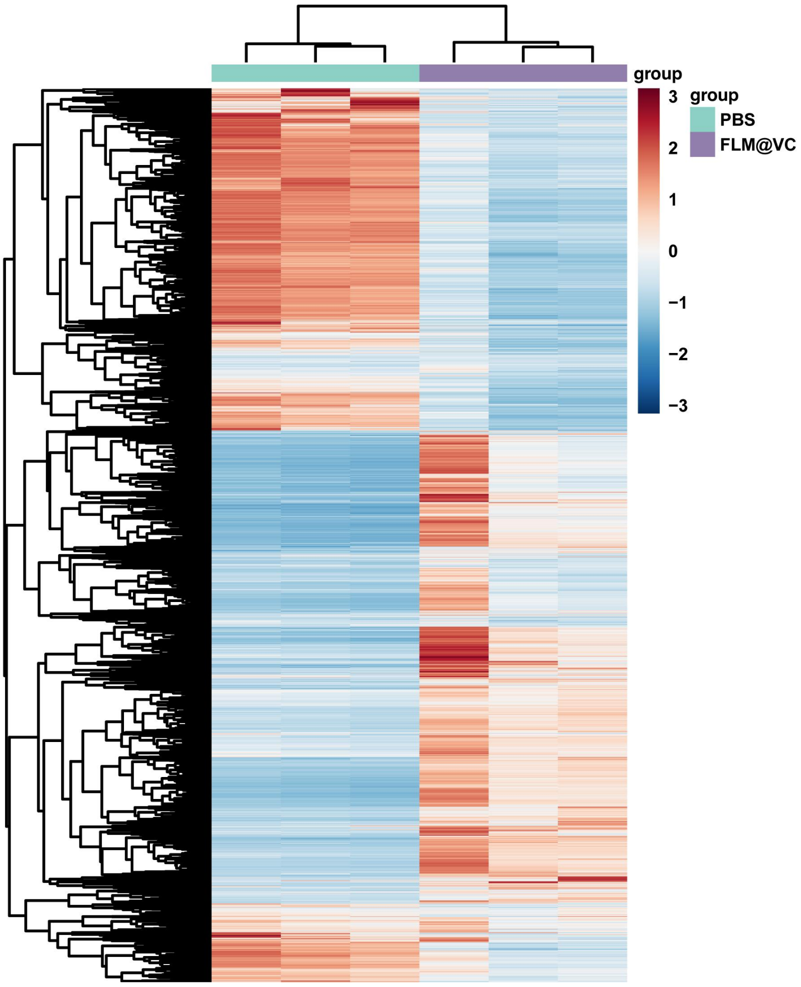

Figure S9. Heat map showing differential expression of genes in the FLM@VC compared with the control group.

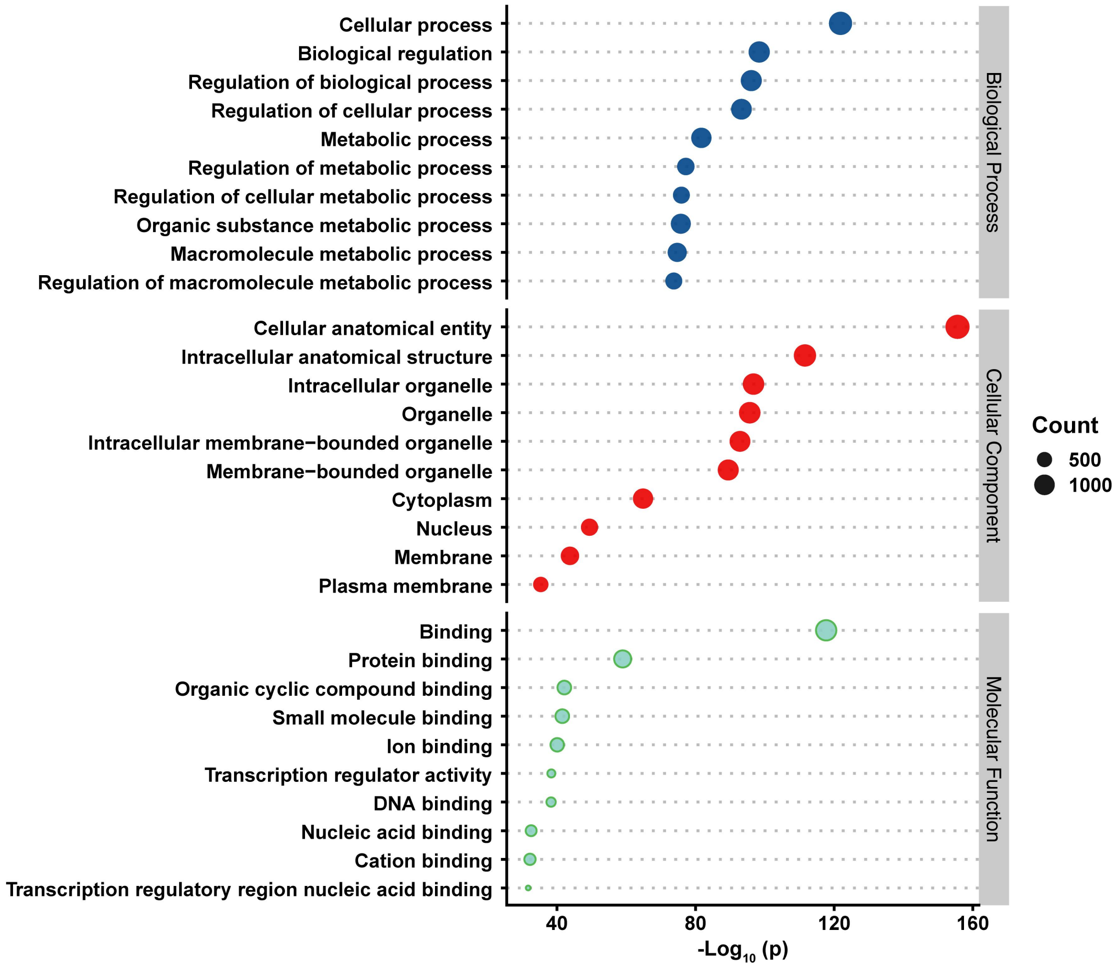

Figure S10. GO annotation analysis in the FLM@VC compared with the control group.

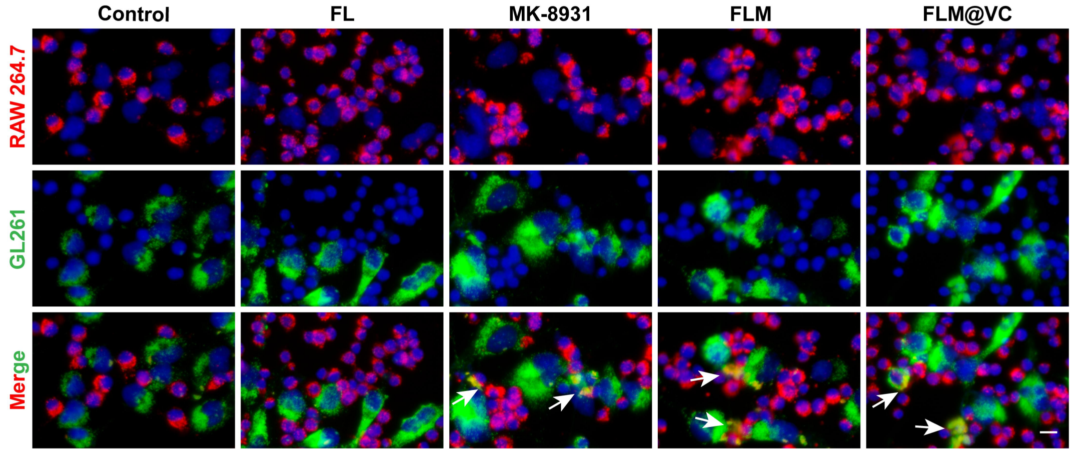

Figure S11. Representative images for analyzing the killing of GL261 tumor cells by the repolarized M1 macrophages, with arrows indicating phagocytosis of tumor cells

by the macrophages (white). Scale bar: 25 μm.

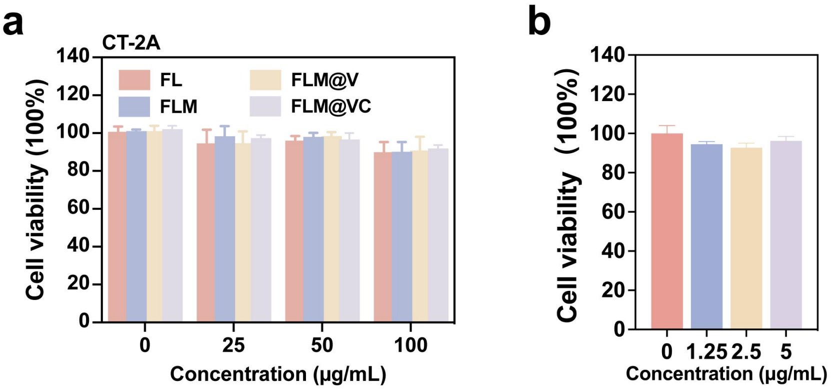

Figure S12. Cell viability of CT-2A cells after co-incubation with FL, MK-8931, FLM, FLM@V and FLM@VC. Data are shown as the mean values ± SD (n = 3).

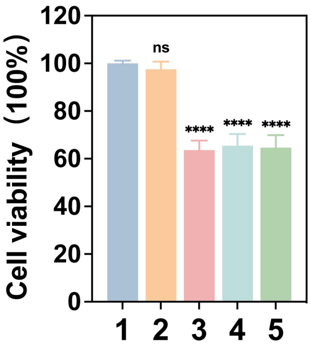

Figure S13. Cell viability of CT-2A cells after various treatments. Data are shown as the mean values ± SD (n = 3). All the statistical significance was analyzed by ANOVA with a Tukey post-hoc test. *P<0.05, **P<0.01, ***P<0.001, and ****P<0.0001, ns, not significant.

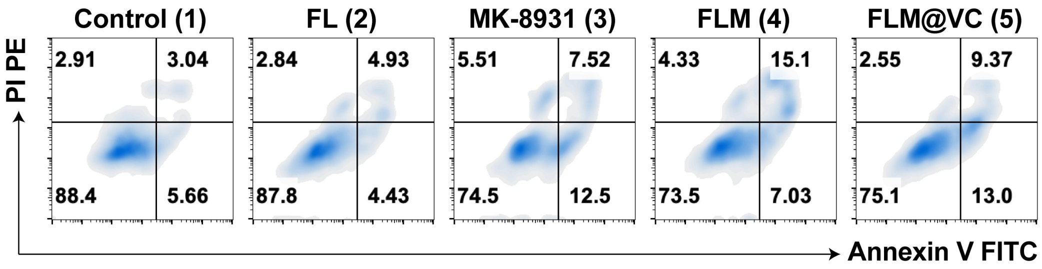

Figure S14. FCM analysis of CT-2A cells apoptosis after various treatments.

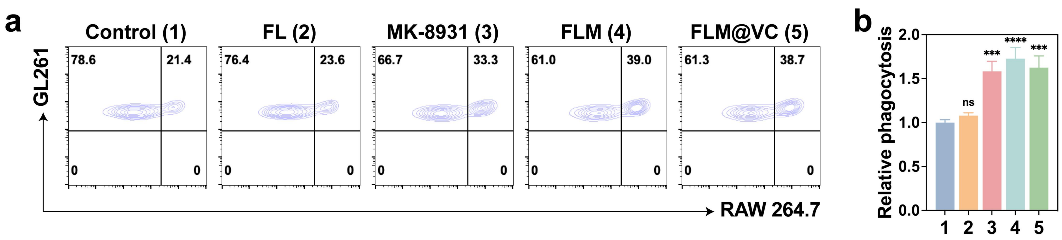

Figure S15. FCM analysis of macrophage phagocytosis of CT-2A cells *in vitro*. Data are shown as the mean values ± SD (n = 3). All the statistical significance was analyzed by ANOVA with a Tukey post-hoc test. *P<0.05, **P<0.01, ***P<0.001, and ****P<0.0001, ns, not significant.

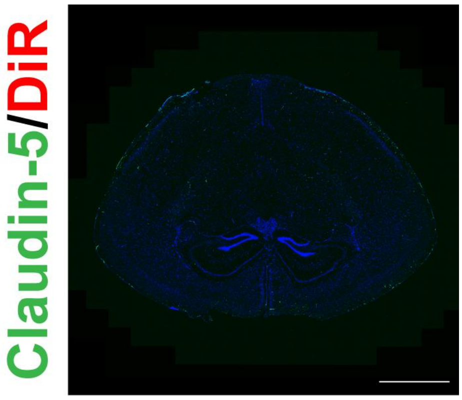

Figure S16. The DiR-labeled FLM@VC distribution in BBB (Scale bar: 2 mm)

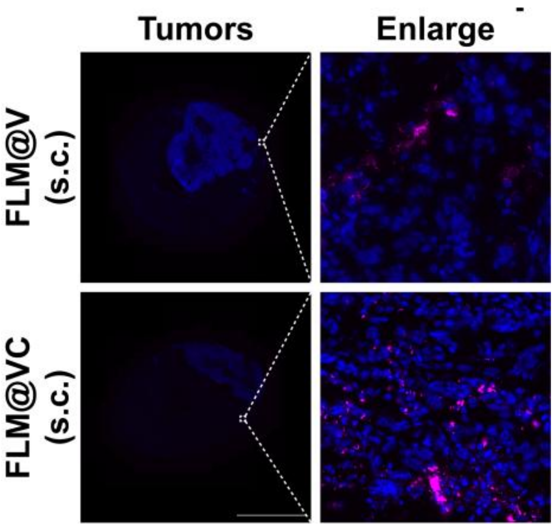

Figure S17. The Cy5-labeled FLM@Vand Cy5-labeled FLM@VC distribution in mouse tumors 24 h after s.c. administration. Scale bar: 5 mm.

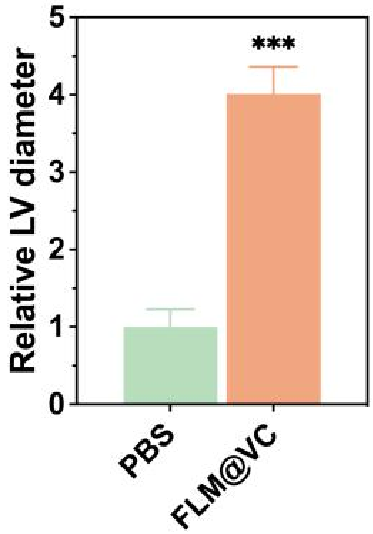

Figure S18. Relative MLV diameter. Data are shown as the mean values ± SD (n = 3). All the statistical significance was analyzed by two-tailed Student’ s t test. *P<0.05, **P<0.01, ***P<0.001, and ****P<0.0001, ns, not significant.

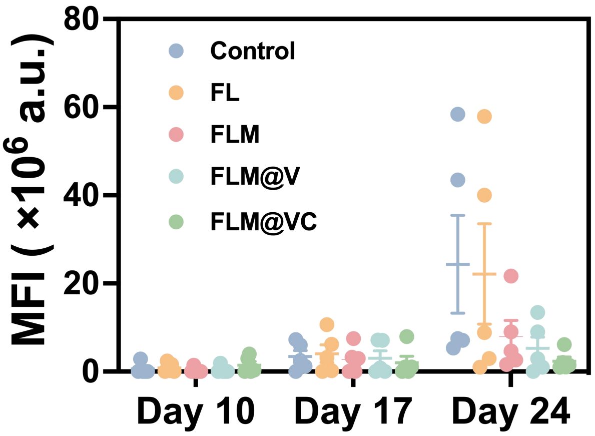

Figure S19. Quantitative bioluminescence images of glioma mice. Data are expressed as the mean ± SD (n = 5).

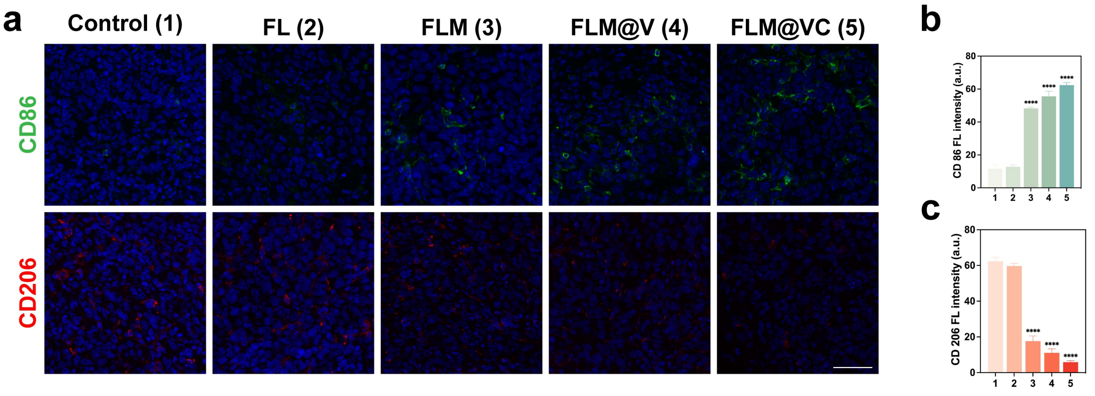

Figure S20. (a) IF staining of CD86 and CD206 in tumor sections after various treatments. Scale bar: 50 μm. (b-c) Quantitative FL analysis of CD86 and CD206. Data are shown as the mean values ± SD (n = 3). All the statistical significance was analyzed by ANOVA with a Tukey post-hoc test. *P<0.05, **P<0.01, ***P<0.001, and ****P<0.0001, ns, not significant.

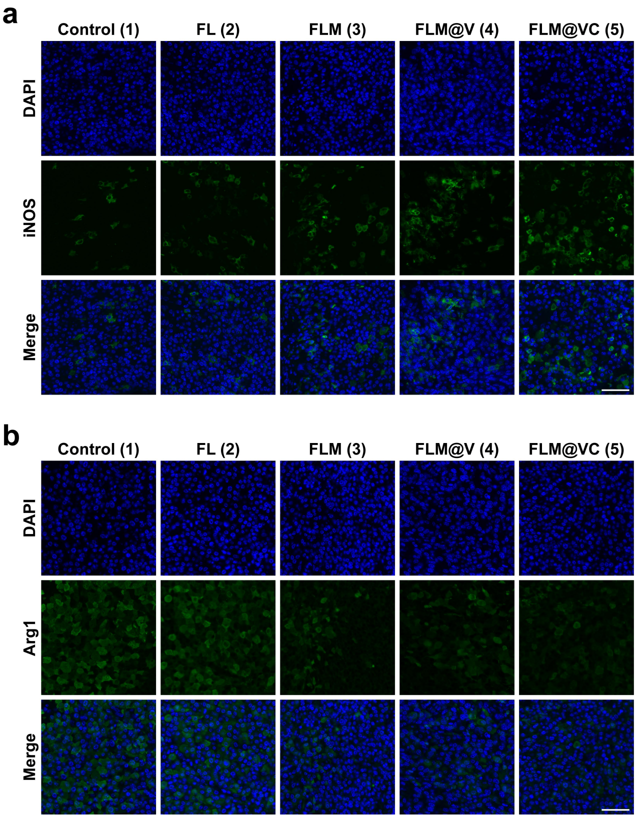

Figure S21. IF staining of (a) iNOS and (b) Arg1 in tumor sections after various treatments. Scale bar: 50 μm.

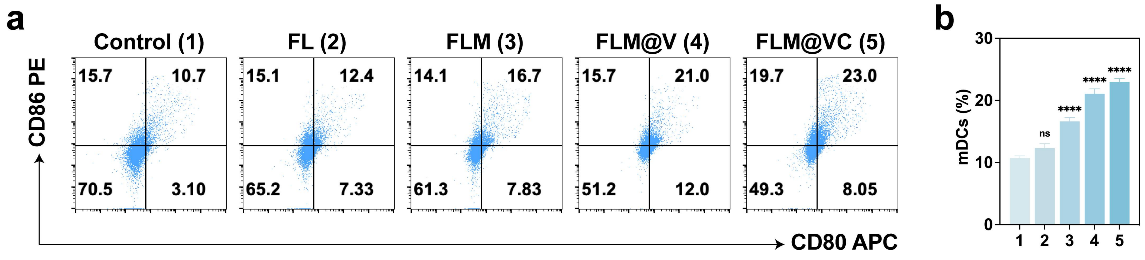

Figure S22. (a)Representative FCM results of matured mDCs within dCLNs after different treatments and (b) the corresponding quantitative analysis. Data are shown as the mean values ± SD (n = 3). All the statistical significance was analyzed by ANOVA with a Tukey post-hoc test. *P<0.05, **P<0.01, ***P<0.001, and ****P<0.0001, ns, not significant.

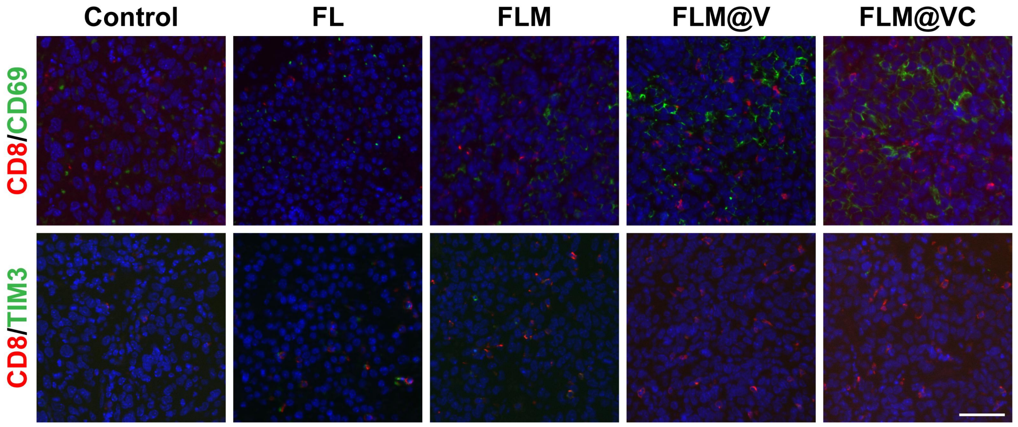

Figure S23. IF staining of $CD8^{+}CD69^{+}$ T cells and $CD8^{+}TIM3^{+}$ T cells in tumor sections after various treatments. Scale bar: 50 μm.

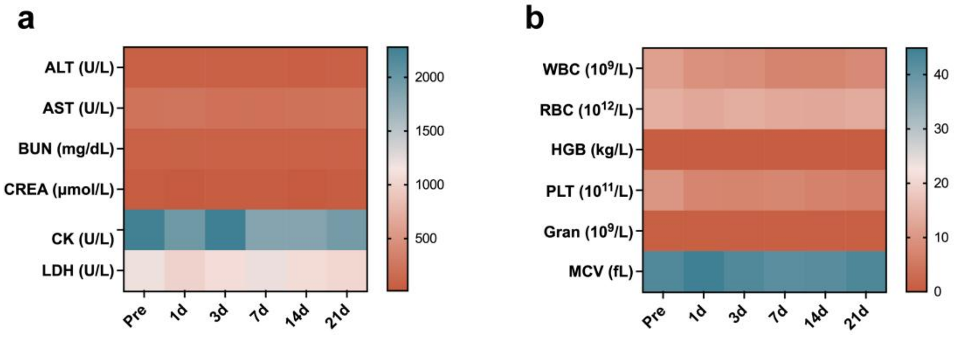

Figure S24. Blood biochemical parameters (a) and blood routine examination (b) of mice 21 days after intravenous injection of FLM@VC. Data are shown as the mean values ± SD (n = 3).

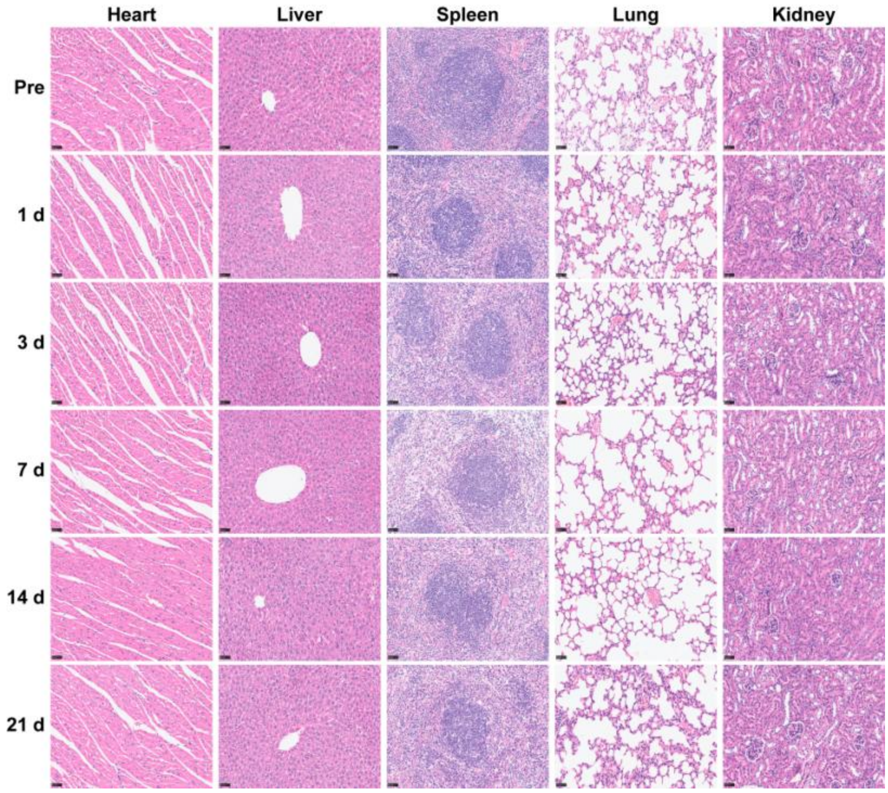

Figure S25. HE staining of the major organs at corresponding time intervals (n = 3). Scale bar: 50 μm.

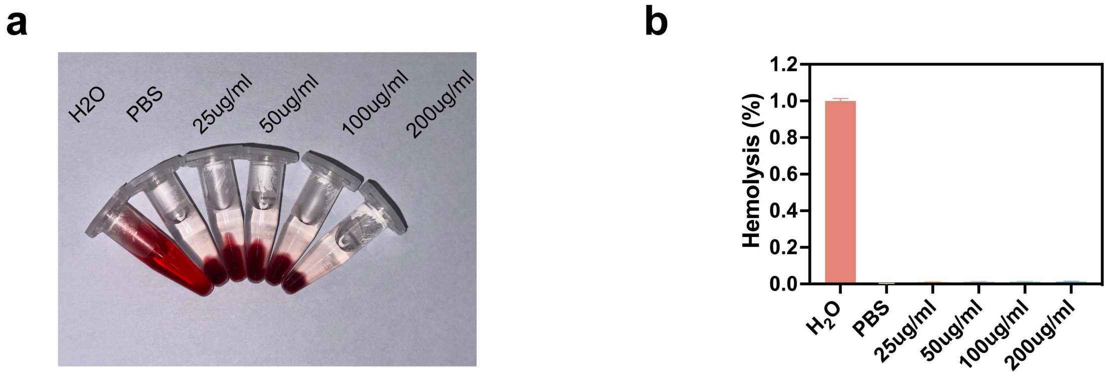

Figure S26. The representative digital photos of hemolysis tests using mouse red blood cells (a) and the corresponding quantitative analysis (b). Data are shown as the mean values ± SD (n = 3).

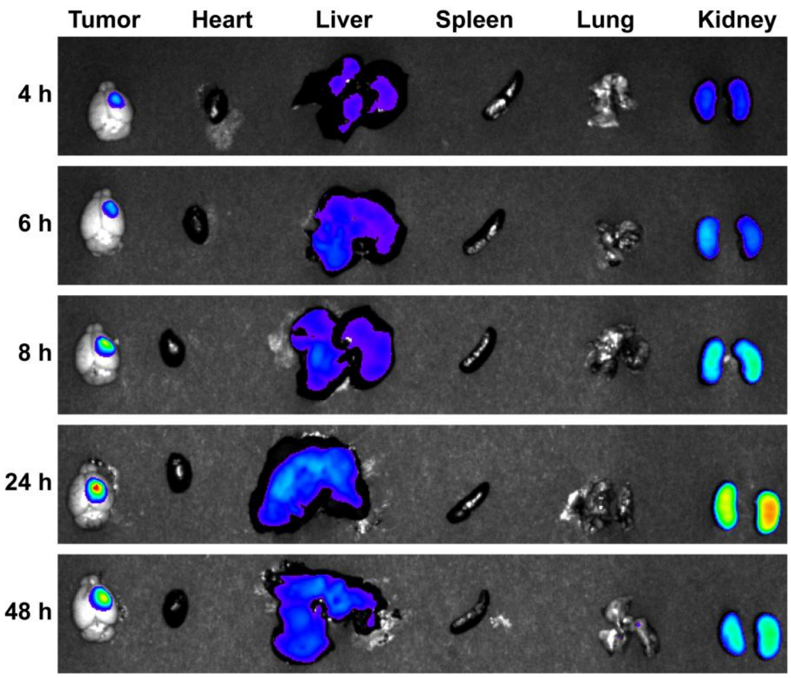

Figure S27. The ex vivo FL images of the extracted tumors and major organs (Spleen, heart, liver, lung, kidney).

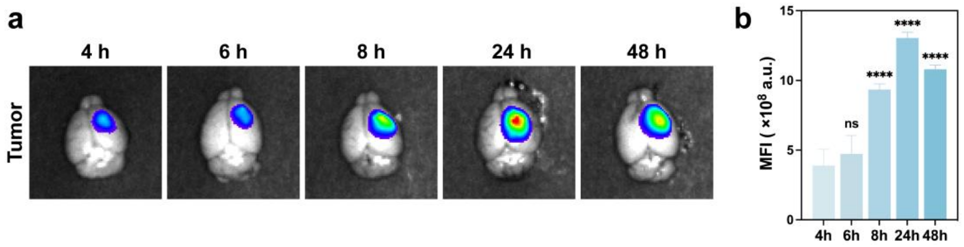

Figure S28. The ex vivo FL images of the extracted tumors at different time points after and quantitative analysis of FL intensity in the tumor. Data are shown as the mean values ± SD (n = 3). All the statistical significance was analyzed by ANOVA. *P<0.05, **P<0.01, ***P<0.001, and ****P<0.0001, ns, not significant.
